# Lactate dehydrogenase B noncanonically promotes ferroptosis defense in *KRAS*-driven lung cancer

**DOI:** 10.1038/s41418-024-01427-x

**Published:** 2024-12-07

**Authors:** Liang Zhao, Haibin Deng, Jingyi Zhang, Nicola Zamboni, Haitang Yang, Yanyun Gao, Zhang Yang, Duo Xu, Haiqing Zhong, Geert van Geest, Rémy Bruggmann, Qinghua Zhou, Ralph A. Schmid, Thomas M. Marti, Patrick Dorn, Ren-Wang Peng

**Affiliations:** 1 Department of General Thoracic Surgery, Inselspital, Bern University Hospital, Bern, Switzerland; 2 Department for BioMedical Research (DBMR), University of Bern, Bern, Switzerland; 3 Second Department of Thoracic Surgery, Hunan Cancer Hospital and The Affiliated Cancer Hospital of Xiangya School of Medicine, Central South University, Changsha, Hunan 410013 China; 4 Department of Biology, Institute of Molecular Systems Biology, Swiss Federal Institute of Technology/ETH Zürich, Zurich, Switzerland; 5 PHRT Swiss Multi-Omics Center, smoc.ethz.ch, Zurich, Switzerland; 6 Department of Thoracic Surgery, Shanghai Chest Hospital, Shanghai Jiao Tong University, Shanghai, China; 7 Lung Cancer Center/Lung Cancer Institute, West China Hospital, Sichuan University, Chengdu, China; 8 Department of Thoracic Surgery, Fujian Medical University Union Hospital, Fuzhou City, Fujian, China; 9 Department of Oncology, The First Affiliated Hospital of Nanjing Medical University, Nanjing, China; 10 Interfaculty Bioinformatics Unit and Swiss Institute of Bioinformatics, University of Bern, Bern, Switzerland; 11 Present Address: Department of Molecular and Integrative Physiology, University of Michigan, Ann Arbor, MI 48109 USA

**Keywords:** Cancer metabolism, Non-small-cell lung cancer

## Abstract

Ferroptosis is an oxidative, non-apoptotic cell death frequently inactivated in cancer, but the underlying mechanisms in oncogene-specific tumors remain poorly understood. Here, we discover that lactate dehydrogenase (LDH) B, but not the closely related LDHA, subunits of active LDH with a known function in glycolysis, noncanonically promotes ferroptosis defense in *KRAS*-driven lung cancer. Using murine models and human-derived tumor cell lines, we show that LDHB silencing impairs glutathione (GSH) levels and sensitizes cancer cells to blockade of either GSH biosynthesis or utilization by unleashing *KRAS*-specific, ferroptosis-catalyzed metabolic synthetic lethality, culminating in increased glutamine metabolism, oxidative phosphorylation (OXPHOS) and mitochondrial reactive oxygen species (mitoROS). We further show that LDHB suppression upregulates STAT1, a negative regulator of SLC7A11, thereby reducing SLC7A11-dependent GSH metabolism. Our study uncovers a previously undefined mechanism of ferroptosis resistance involving LDH isoenzymes and provides a novel rationale for exploiting oncogene-specific ferroptosis susceptibility to treat *KRAS*-driven lung cancer.

## Introduction

Oncogenic *KRAS* mutations are common in non-small cell lung cancer (NSCLC) and other human cancers [1]. Despite advances in targeting KRAS directly or indirectly and the advent of immunotherapy, effective therapies for *KRAS*-mutant NSCLC remain elusive [2]. Mutant KRAS reprograms cancer metabolism [3–6] to meet the increased energetic, biosynthetic and redox demands of tumor cells and promote *KRAS*-induced tumorigenicity [7]. In particular, *KRAS*-mutant cancer has been shown to produce high levels of reactive oxygen species (ROS) and has evolved sophisticated antioxidant programs to overcome the oxidative stress barrier during tumorigenesis [3, 8], on which tumor cells exquisitely depend for survival. Consequently, disruption of ROS defense would be selectively toxic for cancer cells [9, 10].

LDHB (LDH1) and LDHA (LDH2) are subunits of the active tetrameric LDH, which catalyzes the interconversion of lactate/pyruvate and NAD^+^/NADH in glycolysis and plays an important role in ATP generation and energy homeostasis in both anaerobic glycolysis and aerobic glycolysis known as the Warburg effect [11–14]. Despite considerable sequence and structural homology, LDHA and LDHB differ in their subcellular localization and substrate affinities, resulting in distinct functional roles [13, 15–17]. While LDHA predominantly converts pyruvate to lactate, supporting glycolysis under both anaerobic and aerobic conditions, thereby supporting the Warburg effect in cancer cells, LDHB has a higher affinity for lactate, catalyzing its conversion back to pyruvate, which fuels OXPHOS by linking it to the tricarboxylic acid (TCA) cycle [13, 18, 19]. Intriguingly, both LDHA and LDHB have been shown to play critical roles in *KRAS*-mutant cancers, required for tumor progression by regulating mitochondrial activities and stem cell properties, and are viable therapeutic targets for KRAS-dependent NSCLC [20–22]. LDH may also have functions independent of lactate/pyruvate metabolism [13, 17, 23, 24], although the precise mechanisms underlying the role of LDH in cancer remain to be elucidated.

Ferroptosis is an oxidative form of non-apoptotic cell death activated by ROS- and iron-dependent lipid peroxidation of polyunsaturated fatty acids (PUFAs) [25–27], which is often inactivated in cancer [28, 29]. The susceptibility of cancer cells to ferroptosis is finely balanced by the cellular metabolism that triggers lipid oxidation and the antioxidant systems that counteract it [29]. The cystine/glutamate antiporter subunit SLC7A11 (also known as xCT) and the selenium-dependent hydroperoxidase glutathione peroxidase 4 (GPX4) are the most potent antioxidant hubs defending against ferroptosis [30, 31]. Whereas SLC7A11 imports cysteine for GSH biosynthesis, GPX4 utilizes GSH to detoxify lipid peroxides and suppress ferroptosis [32]. Consequently, blockade of the SLC7A11/GPX4 axis with inhibitors (*e.g*. erastin and RSL3) leads to uncontrolled accumulation of lipid peroxides at the plasma membrane and endomembranes, ultimately inducing ferroptosis [29, 32]. Escape from ferroptosis has been shown to contribute to *Kras*-driven tumor development and progression [33, 34]. However, the antioxidant adaptations specific to oncogenic KRAS, such as the cellular processes that impinge on key antioxidant proteins and thereby modulate ferroptosis sensitivity of *KRAS*-dependent NSCLC, remain poorly understood [35].

In this study, we report for the first time that LDHB, but not LDHA, plays a role in protecting *KRAS*-mutant NSCLC from ferroptosis. LDHB modulates GSH metabolism through a noncanonical role in the regulation of SLC7A11, and as a result, LDHB suppression sensitizes cancer cells to SLC7A11/GPX4 inhibition by unleashing ferroptosis-mediated synthetic lethality in vitro and in vivo, which is mechanistically driven by increased glutamine metabolism, OXPHOS and mitoROS. Our results reveal a novel mechanism of ferroptosis defense involving LDH isoenzymes and provide a viable rationale for exploiting oncogene-specific ferroptosis susceptibility to treat *KRAS*-mutant lung cancer.

## Results

### LDHB silencing impairs GSH metabolism in *KRAS*-dependent lung cancer cells

**Fig. 1:**
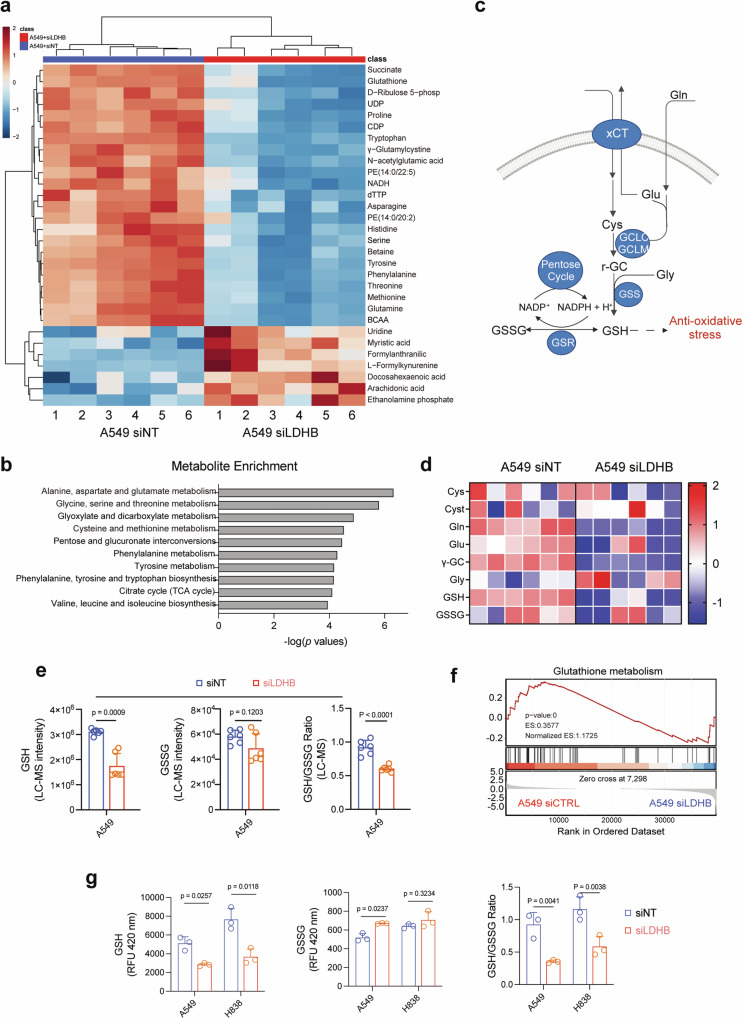
LDHB silencing impairs GSH metabolism in *KRAS*-dependent NSCLC cells. **a** Metabolomic analysis (LC-MS) of LDHB KD (siLDHB) and control (siNT) A549 cells (48 h post-transfection). Heat map showing the top 30 metabolites significantly different between LDHB KD and control A549 cells (*n* = 12). Relative abundance is scaled between 2 to -2. **b** Pathway enrichment analysis shows significantly downregulated metabolic processes in LDHB KD compared to control A549 cells. **c** Schematic of de novo GSH synthesis and the effect of LDHB KD on the pathway. Highlighted in blue are the genes and metabolites significantly altered by LDHB KD in A549 cells. Cys, cysteine; Cysta, cystathionine; γ-GC, γ-glutamylcysteine; Gly, glycine; Gln, glutamine; Glu, glutamate; GSH, glutathione; GSSG, glutathione disulfide; GCLC/GCLM, glutamate-cysteine ligase (GCL) catalytic and modifier subunits; GSS, glutamine synthetase; GSR, glutathione reductase; xCT, SLC7A11. **d** Heat map illustrating the abundance of key GSH metabolites in LDHB KD and control A549 cells. **e** The abundance of GSH, GSSG, and GSH/GSSG ratio in LDHB KD and control A549 cells. The analysis was based on the LC-MS data of A549 cells, *p* values by Student’s *t*-test. **f** LDHB KD downregulates GSH metabolism gene signature. GSEA was based on the transcriptome of LDHB KD (siLDHB) and control (siNT) A549 cells. **g** Ratios of GSH/GSSG in LDHB KD and control cells transfected with siRNAs for 48 h. Data are shown as mean ± s.d. (*n* = 3), with *p* values by Student’s *t*-test.

We have recently shown that LDHB plays an important role in tumor-initiating cells and targeting LDHB affects mitochondrial metabolism in NSCLC [20]. To better understand the metabolic pathways underlying LDHB function in *KRAS*-driven NSCLC [20, 22], we performed unbiased metabolomics of A549 cells using LC-MS. LDHB knockdown (KD) by siRNAs significantly downregulated a number of cellular metabolites enriched in multiple metabolic processes (Fig. 1a, b; Fig. S1a; Original data file 1). LDHB KD A549 cells also showed reduced levels of intracellular lactate (Fig. S1b), consistent with its role in the Warburg effect [12, 14]. In particular, several intermediate metabolites of the de novo synthesis of GSH (Fig. 1c), such as cysteine (Cys), γ-glutamylcystine (γ-GC), glutamine (Gln), glutamate (Glu), and GSH itself, as well as the ratio of GSH to glutathione disulfide (GSSG), the oxidized form of GSH, were significantly decreased upon LDHB KD, although GSSG per se was not significantly altered in LDHB KD A549 cells (siLDHB) compared to control A549 cells (siNT) (Fig. 1d, e). In support of the metabolomics results, re-analysis of our transcriptomic data from A549 cells [20] showed that LDHB silencing significantly reduced the GSH gene signature and the mRNA levels of several key enzymes involved in GSH synthesis (Fig. 1c; Fig. 1f; Fig. S1c). Importantly, we confirmed that LDHB KD in *KRAS*-dependent A549 and H838 cells (Table S1) significantly reduced the GSH/GSSG ratio, a standard measure of cellular oxidative stress (Fig. 1g), but did not significantly alter total ROS in LDHB KD compared to control A549 cells (Fig. S1d), consistent with the metabolomics results (Fig. 1e). These results indicate that LDHB silencing impairs GSH metabolism, suggesting a novel role for LDHB in regulating antioxidant programs in *KRAS*-dependent NSCLC.

### LDHB suppression sensitizes *KRAS*-dependent NSCLC cells to blockade of GSH-dependent ferroptosis defense

**Fig. 2:**
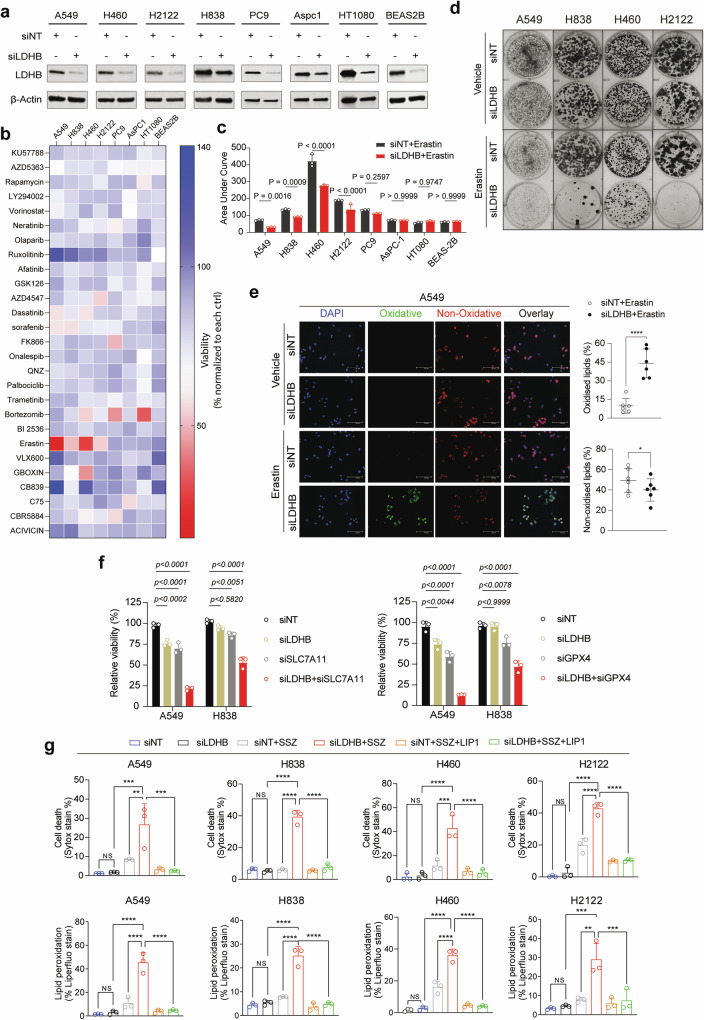
LDHB deficiency sensitizes *KRAS*-mutant NSCLC cells to ferroptosis inducers. **a** Immunoblots of the indicated cells transfected with siNT or siLDHB for 48 h. **b** Heat map showing relative viability of LDHB KD cells treated for 72 h with the indicated compounds dosed at IC_80_/IC_90_ in control cells. Data are expressed as percentages of viable LDHB KD cells normalized to the corresponding control cells. **c** Sensitivity of LDHB KD cells and control cells to erastin dosed at IC_80/90_ in control cells. Drug sensitivity is determined by the area under curve (AUC) calculated by Graphpad 9.1. Data are presented as mean ± s.d. (*n* = 3), with *p* < 0.05 by Student’s *t*-test. **d** Clonogenic assay of the indicated human *KRAS*-mutant NSCLC cells transfected with siLDHB and siNT and treated for 72 h with erastin (A549, 1 μM; H838, 0.25 μM; H460, 10 μM; H2122, 5 μM) or DMSO. **e** A549 cells transfected with siNT or siLDHB for 36 h were treated with DMSO or erastin (5 μM) for another 14 h before stained with C11 BODIPY 581/591. Scale bars, 100 μm. **f** Viability assay of A549 and H838 cells transfected with siNT or siLDHB for 24 h and subsequently transfected with siNT, siSLC7A11 or siGPX4 for additional 48 h. Data are shown as mean ± s.d. (n = 3), with the statistical analyses by one-way ANOVA. ns, no significant difference. **g** Cell death and lipid peroxidation assay of the indicated cells transfected with siNT and siLDHB for 48 h followed by further treatment for 16 h with sulfasalazine (SSZ; A549, 1 mM; H838, 0.5 mM; H460, 2.5 mM; H2122, 2 mM), alone or with Liproxstatin-1 (LIP1; 3 µM). Data are shown as mean ± s.d. (*n* = 3). ** *p* < 0.01; ***p < 0.001; ****p < 0.0001 by one-way ANOVA.

GSH is produced by the two-step synthesis of a tripeptide L-glutamic acid, cysteine, and glycine, with the cysteine required for GSH synthesis being obtained by SLC7A11-mediated uptake. We therefore hypothesized that LDHB KD would induce dependence on the SLC7A11/GSH antioxidant program. Indeed, a synthetic lethal chemical screen using small molecule inhibitors (*n* = 22) targeting multiple metabolic and oncogenic pathways, including the SLC7A11 inhibitor erastin (Table S2), showed that erastin and to a lesser extent sorafenib and dasatinib, preferentially suppressed the viability of LDHB KD lung cancer cells (A549, H838, H460 and H2122) harboring *KRAS* alterations, as measured by their AUC (area under curve) decrease in LDHB KD cells compared to control cells (Original data file 2**)**. Interestingly, the increased susceptibility to erastin upon LDHB depletion was only observed in *KRAS*-dependent NSCLC cells, but not in *EGFR*-mutant PC9 (NSCLC), *KRAS*-mutant AsPC1 (pancreatic cancer), HT1080 (fibrosarcoma) or BEAS-2B, a normal epithelial cell line (Fig. 2a–c), suggesting that the antioxidant role of LDHB is oncogene- and lineage-specific. Confirming this finding, LDHB suppression sensitized *KRAS*-mutant A549, H838, H460 and H2122 cells to erastin in clonogenic assay (Fig. 2d), which was accompanied by a significant increase in oxidized lipids (Fig. 2e). Moreover, LDHB KD significantly increased the sensitivity of A549 cells to genetic inhibition of not only SLC7A11 but also GPX4 (Fig. 2f), which utilizes GSH to detoxify lipid peroxidation, further supporting a GSH-dependent role for LDHB. In sharp contrast, LDHB silencing (siRNA) failed to sensitize several *KRAS* wild-type NSCLC cell lines (H1299, H522 and Calu-3) to erastin (Fig. S2a).

Similar results were obtained with sulfasalazine (SSZ), an FDA-approved drug with ferroptosis-inducing activity via inhibition of cystine/glutamate antiporter (xCT), which induced a significantly greater cell death and lipid peroxidation in LDHB KD A549, H838, H460 and H2122 cells than in control cells (Fig. 2g). Importantly, SSZ-induced cell death and lipid peroxidation in LDHB KD cells could be robustly reversed by liproxstatin-1 (LIP1) (Fig. 2g), a ferroptosis inhibitor that eliminates lipid hydroperoxides and has an anti-ferroptotic effect similar to GPX4 [30, 32], suggesting that SSZ-induced synthetic lethality in LDHB KD cells is mediated by ferroptosis.

**Fig. 3:**
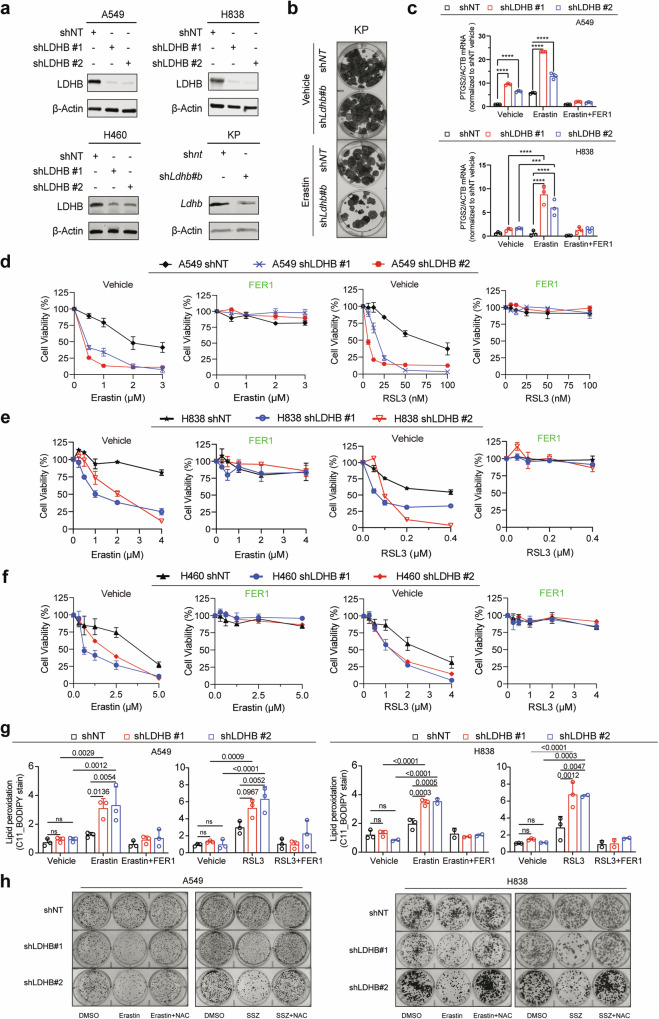
Inhibiting LDHB and the SLC7A11/GSH/GPX4 axis confers ferroptosis-mediated metabolic synthetic lethality. **a** Immunoblot of the indicated cells stably transduced with shNT or shRNAs against LDHB. **b** Clonogenic assay of murine KP cells transduced with sh*Ldhb#b* and shNT and further treated for 72 h with erastin (15 μM) or DMSO. **c** Quantitative analysis (qPCR) of *PTGS2* mRNA in the indicated cells treated for 14 h with DMSO or erastin (A549, 5 µM; H838, 2.5 µM) with or without FER1 (3 µM). **d–f** Viability assay of shNT- or shLDHB-transduced cells after treated with RSL3 or erastin, alone or in combination with 2 μM Ferrostatin-1(FER1). **g** Flow cytometry of C11 BODIPY mean fluorescence intensity ratio of oxidative channel (FITC 488 nm) versus non-oxidative channel (PE-TEXAS RED 610 nm) in shNT- or shLDHB-transduced cells. A549 cells were treated with 0.5 μM RSL3 or 5 μM erastin, alone or in combination with 2 μM FER1 for 6 h and 14 h, respectively. H838 cells were treated with 0.25 μM RSL3 or 2.5 μM erastin, alone or in combination with 2 μM FER1 for 6 h and 14 h, respectively. Data are shown as mean ± s.d (n = 3), with statistical analyses by two-way ANOVA. **h** Colony assay of A549 and H838 shNT- or shLDHB-transduced cells treated with erastin (A549, 5 µM; H838, 2.5 µM) or sulfasalazine (SSZ; A549, 0.25 mM; H838, 1 mM) for 24 h in the presence or absence of NAC (10 mM).

This finding was validated by several independent assays in which shRNA-mediated KD of LDHB (Fig. 3a) sensitized A549, H838 and murine KP (*Kras*^*G12D/+*^; *p53*^*-/-*^) cells to erastin and the GPX4 inhibitor RSL3, accompanied by a significant increase in *PTGS2* (prostaglandin endoperoxide synthase 2) expression, a ferroptosis biomarker [36], and lipid peroxidation (Fig. 3b–g). Importantly, the erastin- and RSL3-induced upregulation of PTGS2, loss of cell viability and increase in lipid peroxidation in LDHB KD cells were almost completely rescued by FER1, but not by inhibitors of necrosis (NEC), autophagy (HCQ) or apoptosis (ZVF) (Fig. 3c–g; Fig. S2b–d). Notably, the antioxidant N-acetylcysteine (NAC), a ROS scavenger, could also largely attenuate the erastin- or SSZ-induced suppression of cell viability in LDHB KD cells (Fig. 3h). These results accommodate evidence for a novel role of LDHB in GSH-associated ferroptosis denfense, and its suppression sensitizes KRAS-dependent NSCLC cells to blockade of the SLC7A11/GSH/GPX4 antioxidant program.

### Distinct roles of LDHA and LDHB in *KRAS*-driven NSCLC

Like LDHB, LDHA is required for the Warburg effect [35] and promotes tumor cell survival by protecting against ROS [18, 37]. Importantly, LDHA has been shown to play an essential role in *KRAS*-driven NSCLC, as a lack of LDHA results in reduced tumorigenesis, disease regression, reprogramming of pyruvate metabolism and a reduction in lactate accumulation in a mouse model of *Kras*-mutant NSCLC [21]. Furthermore, LDHA activity is important for the development of *RAS*-induced fibrosarcoma [38]. We therefore tested whether LDHA has a similar function to LDHB in the regulation of ferroptosis. However, in sharp contrast to the LDHB scenario, siRNA-based LDHA KD (Fig. S3a) and inhibitors (GSK2837808A, R-GNE-140) of LDHA showed no apparent effect on the sensitivity of A549 cells to RSL3 or erastin (Fig. S3b-d). To rule out a cell line-specific effect, we further tested *KRAS*-driven H838 and H460 cells and found similar results to A549 cells (Fig. S3e). Thus, LDHA, although closely related to LDHB, is not involved in the control of ferroptosis, suggesting that the two LDH isoenzymes have distinct roles in *KRAS*-mutant NSCLC.

### LDHB knockdown suppresses SLC7A11 expression through the upregulation of STAT1

**Fig. 4:**
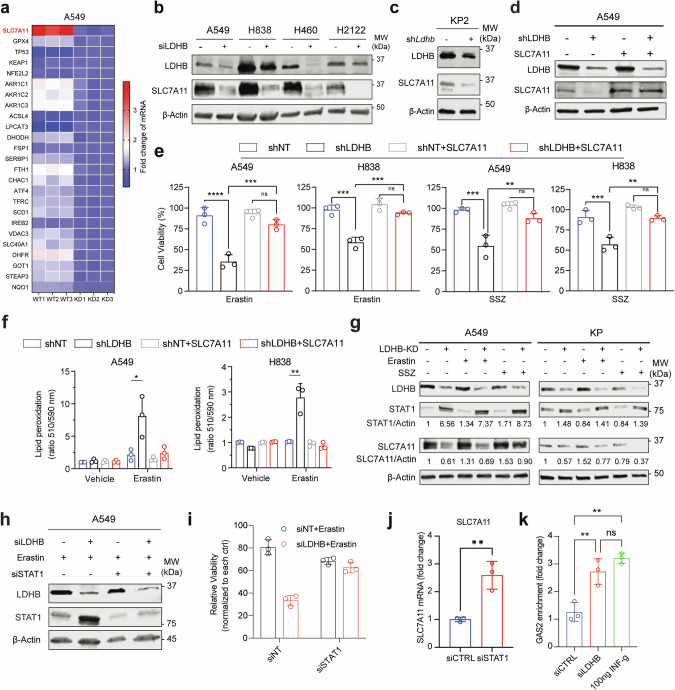
LDHB regulates GSH-dependent ferroptosis defense through SLC7A11. **a** Heat maps showing the mRNA fold change of ferroptosis-related genes (*n* = 25) in siLDHB cells (KD) compared to siNT cells (WT) (based on RNA-seq data). **b, c** Immunoblots of the indicated cells transfected for 48 h with siNT (-) or siLDHB (+) or stably expressing shRNAs. **d** Immunoblot of A549 stably expressing shNT or shLDHB were further transduced with either an empty vector or a SLC7A11-expressing plasmid (pCMV-SLC7A11). **e, f** Viability and lipid ROS assay of the indicated cells treated with erastin (A549, 5 µM; H838, 2.5 µM) or sulfasalazine (SSZ; A549, 0.25 mM; H838, 1 mM) for 24 h. **g** Immunoblots of A549 transfected (48 h) with siLDHB or siNT and further treated (16 h) with erastin (5 uM) or Sulfasalazine (1 mM). Murine KP cells expressing Ldhb shRNA or control shRNA were treated with erastin (10 uM) or Sulfasalazine (1 mM) for 24 h. **h, i** Immunoblots **h** and viability assay **i** of A549 cells transfected with siLDHB, siSTAT1 or siNTs for 24 h, followed by further treatment with erastin (10 µM) for another 24 h. **j** SLC7A11 mRNA fold change in A549 cells transfected (72 h) with siSTAT1 compared to A549 cells transfected with siNT. **p < 0.01 by Student’s *t*-test. **k** ChIP of STAT1 in A549 cells transfected with siLDHB or siNT, or treated with IFNγ. STAT1 binding to the SLC7A11 promoter (GAS2 domain) was quantified by qPCR. Results are expressed as fold change in site occupancy over IgG control and shown as mean ± SD from three independent experiments (*n* = 3). **p < 0.01 by two-way ANOVA.

Next, we investigated the mechanism by which LDHB promotes GSH-associated ferroptosis defense. Re-analysis of our transcriptomic data from A549 cells [20] revealed that siRNA-mediated KD of LDHB most significantly downregulated SLC7A11 among other ferroptsosis-related genes (Fig. 4a), which we independently confirmed at the protein level: LDHB KD reduced the SLC7A11 protein in A549, H838, H460, H2122 and murine KP (*Kras*^*G12D/+*^*; p53*^*-/-*^) cells (Fig. 4b, c), as well as in A549 xenografts carrying LDHB-targeting shRNAs (Fig. S4a). Importantly, forced overexpression of SLC7A11 almost completely overcame the erastin- and SSZ-induced suppression of cell viability in LDHB KD A549 and H838 cells, accompanied by a significant decrease in lipid peroxidation (Fig. 4d–f). These results suggest that LDHB promotes GSH-associated ferroptosis defense by regulating SLC7A11, supporting a role for LDHB beyond glycolysis in *KRAS*-mutant NSCLC.

Recent studies have shown that some metabolic enzymes have non-canonical functions to promote cancer progression, in addition to their known roles in metabolism [39]. Notably, our RNAseq data revealed that LDHB KD significantly upregulated the interferon α/γ (IFNα/γ) response genes in A549 cells [20]. Similar results were observed in erastin-treated A549 cells, where the IFNα/γ pathway was among the top candidates most significantly upregulated in erastin-treated LDHB KD vs. erastin-treated control A549 cells (Fig. S4b, c; Original data file 3). IFNα/γ and the downstream STAT1 promote ferroptosis through transcriptional repression of SLC7A11 [40], and we confirmed that LDHB KD alone and in combination with erastin or SSZ markedly increased STAT1 protein levels and concomitantly decreased SLC7A11 in A549, H358, and murine KP cells, whereas erastin or SSZ alone had no effect on STAT1 expression (Fig. 4g, h). STAT3, SCD1, C-MYC and ACSL4, previously shown to contextually regulate ferroptosis, were also not affected under the same conditions (Fig. S4d). Interestingly, we observed that LDHB KD, but not its combination with erastin, slightly increased the protein gasdermin D (GSDMD) in A549 cells (Fig. S4e), which is a key mediator of inflammasome-dependent pyroptotic cell death [41, 42].

Importantly, siRNA KD of STAT1 reversed and largely overcame the inhibition of erastin on the viability of LDHB KD A549 cells (Fig. 4i), suggesting that STAT1 activity is functionally required and sufficient for erastin-induced ferroptosis in LDHB KD cells. STAT1 KD significantly upregulated *SLC7A11* mRNA levels (Fig. 4j), consistent with previous findings that STAT1 transcriptionally represses SLC7A11 [43]. Moreover, STAT1 chromatin immunoprecipitation (ChIP) followed by quantitative PCR (qPCR) demonstrated the enrichment of GAS – an element within the *SLC7A11* promoter that physically interacts with STAT1 and is associated with IFNγ-mediated *SLC7A11* transcriptional repression [43] - when STAT1 antibodies were used, compared to IgG control (Fig. 4k). Notably, LDHB KD further enhanced STAT1 occupancy at the GAS2 site (Fig. 4k), mirroring the effect seen with IFNγ treatment (Fig. 4k). These findings confirm that STAT1 directly binds the SLC7A11 promoter and indicate that LDHB modulates the STAT1/GAS interaction. Collectively, these results suggest that LDHB KD downregulates SLC7A11 expression via the upregulation of STAT1.

### LDHB/SLC7A11 inhibition induces ferroptosis by activating glutamine metabolism

**Fig. 5:**
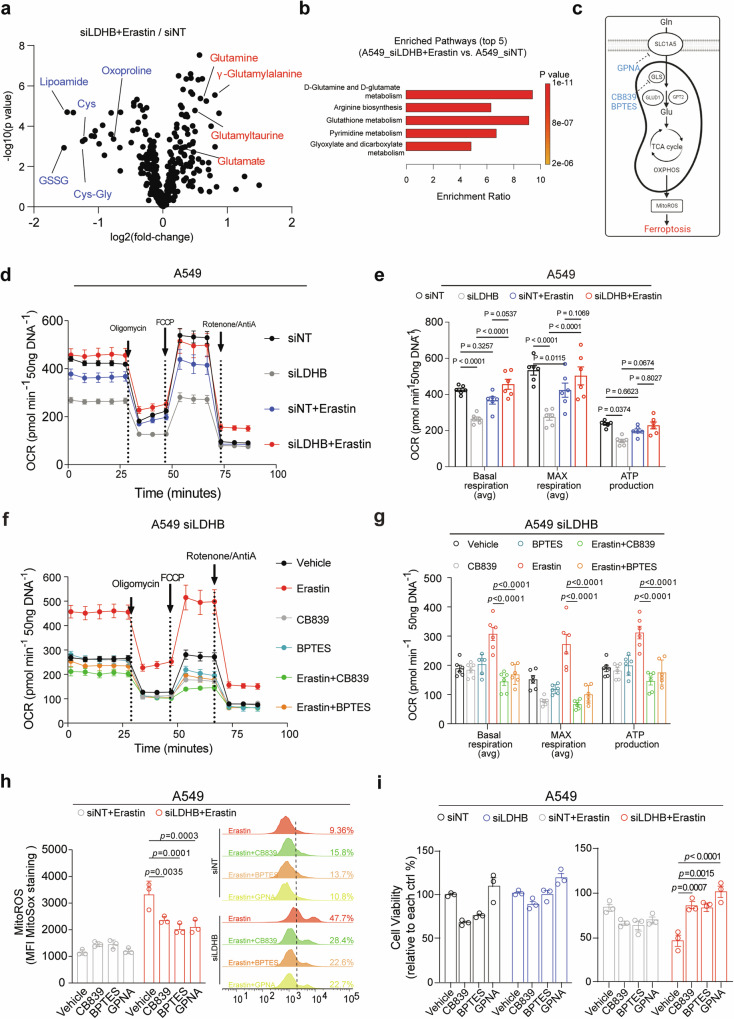
Metabolic synthetic lethality induced by LDHB/SLC7A11 inhibition converges on glutamine metabolism. **a** Volcano plot showing the metabolomics profile of A549 cells transfected for 48 h with siLDHB or siNT and further treated with erastin (5 µM) or vehicle for 20 h. **b** The metabolic pathways most significantly upregulated in erastin-treated LDHB KD cells compared to those in vehicle-treated control cells. **c** Schematic of glutamine metabolism and glutamine-fueled glutaminolysis. Inhibitors that mitigate LDHB/SLC7A11 inhibition-induced ferroptosis are highlighted in green. **d, e** Oxygen consumption rate (OCR) measurement **d** and quantification **e** of A549 cells transfected with siLDHB or siNT for 48 h and further treated for 20 h with DMSO or erastin (5 μM). Cell numbers normalized to 50 ng/DNA. Data represent the average of basal respiration, maximal respiration, and ATP production taken at multiple time points during the respective phases of the Seahorse assay, and are shown as mean ± s.e.m (*n* = 6). **f, g**, OCR measure **f** and quantification **g** of A549 KD cells transfected with siLDHB or siNT for 48 h and further treated for 20 h with DMSO, erastin (5 μM), CB839 (0.5 μM), BPTES (2 μM), and GPNA (50 μM), alone or in combination. Normalization was based on vehicle-treated siNT and siLDHB groups (set as 100%), respectively. Data represent the average of basal respiration, maximal respiration, and ATP production taken at multiple time points during the respective phases of the Seahorse assay, and are shown as mean ± s.e.m (*n* = 6). **h** MitoSox (mitochondrial ROS marker) quantification (left) and flow cytometry (right) analysis of A549 cells transfected and treated as above **d–g**; MitoROS is shown as mean fluorescence intensity (MFI) ± s.d. (*n* = 3), with the siNT group used for normalization. **i** Viability assay of A549 cells transfected with siLDHB or siNT for 48 h and further treated for 20 h with DMSO, erastin (5 μM), CB839 (0.5 μM), BPTES (2 μM), and GPNA (50 μM), alone or in combination. Normalization was based on vehicle-treated siNT and siLDHB groups (set as 100%), respectively.

To elucidate the metabolic process underlying ferroptosis upon LDHB/SLC7A11 inhibition, we performed metabolomic analysis and found that erastin inhibits glutathione metabolism but activates glutamine metabolism in LDHB KD cells, evidenced by a significant decrease in cysteine (Cys), cysteinylglycine (Cys-Gly) and GSSG but an increase in intracellular glutamine and glutamate in LDHB KD A549 cells compared to control A549 cells (Fig. 5a–c; Original data file 4), in agreement with our results from LDHB KD cells (Fig. 1). Notably, erastin-treated LDHB KD A549 cells also showed a significant accumulation of γ-glutamyl-peptides such as glutamylalanine and L-glutamyltaurine (Original data file 4), in line with the recent finding that cysteine deprivation promotes the synthesis of γ-glutamyl peptides due to a non-canonical activity of the glutamate-cysteine ligase catalytic subunit (GCLC) [44].

Glutamine and glutamine-fueled glutaminolysis play a versatile role in cellular metabolism to provide glutamate for the tricarboxylic acid (TCA) cycle and GSH biosynthesis, thereby orchestrating mitochondrial oxidative phosphorylation (OXPHOS), a major source of mitochondrial ROS [45]. Both glutaminolysis and mitoROS have been shown to be crucial for the execution of ferroptosis [46]. Accordingly, we analyzed the real-time oxygen consumption rate (OCR) to assess mitochondrial OXPHOS activity. As expected, LDHB KD alone significantly decreased OCR, consistent with LDHB’s well-known role in mitochondrial function [13, 15, 20]. However, in erastin-treated LDHB KD cells, we observed a significant increase in OCR compared to the siLDHB group (Fig. 5d, e). Notably, pharmacological inhibition of GLS (CB839; BPTES) or SLC1A5 (GPNA), which suppresses glutamine uptake and subsequent glutaminolysis (Fig. 5c), significantly abrogated the erastin-induced increase in OCR (Fig. 5f, g) and mitoROS (Fig. 5h) in LDHB KD cells, but not in control A549 cells (Fig. S5a, b). Importantly, this decrease in OCR and mitoROS was accompanied by an attenuated toxicity of erastin, as measured by a significant rescue of cell viability suppression in erastin-treated LDHB KD cells but not in erastin-treated control A549 cells (Fig. 5i), although the inhibitors alone had no or only a mild effect on the viability of LDHB KD or control A549 cells (Fig. 5i). Thus, the erastin-induced increase in OCR and mitoROS in LDHB KD cells is driven by enhanced glutaminolysis, and these metabolic alterations are directly linked to ferroptotic cell death.

In summary, our results suggest that the inhibition of LDHB and SLC7A11 induces ferroptosis-dependent metabolic synthetic lethality by activating glutamine metabolism. This metabolic shift fuels mitochondrial OXPHOS and increases mitoROS production, creating a heightened dependency on antioxidant defenses. However, due to limited GSH availability, this defense mechanism fails, ultimately resulting in ferroptotic cell death.

### In vivo effects of LDHB/SLC7A11 inhibition in *KRAS*-driven NSCLC

**Fig. 6:**
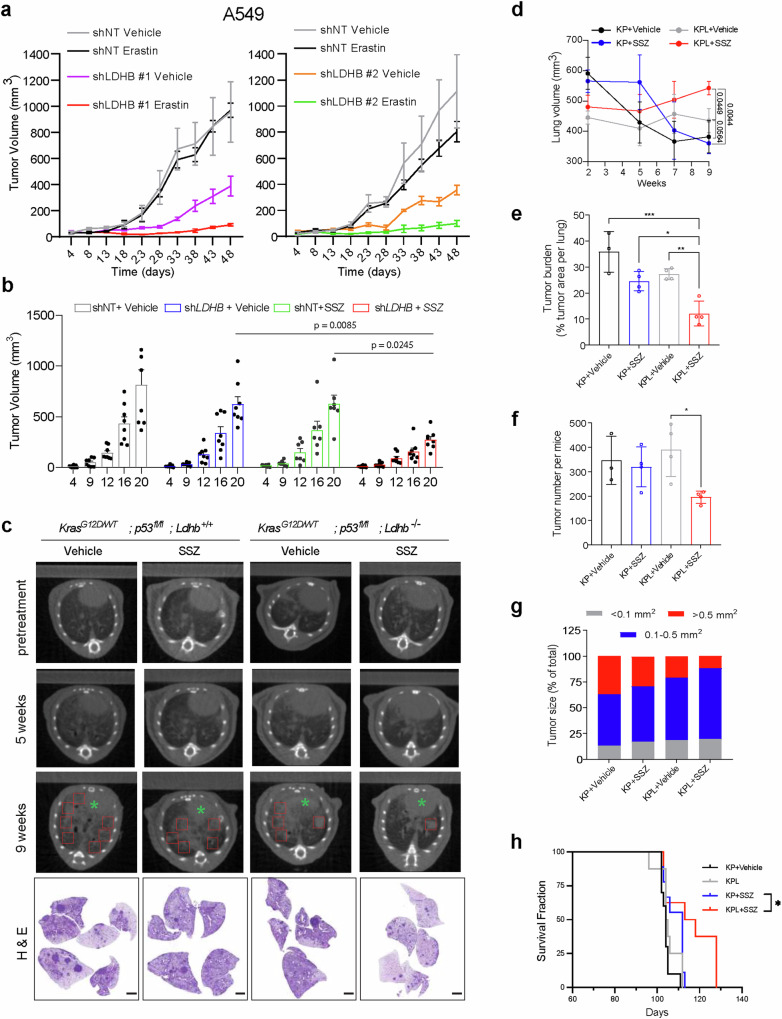
SLC7A11 inhibitors suppress in vivo growth of *LDHB*-deficient *KRAS*-driven lung tumors. **a, b** Tumor development of A549 **a** and H460 **b** xenografts treated with erastin (30 mg/kg/day) and SSZ (150 mg/kg/day). **c** Micro-CT images of *LSL-Kras*^*G12D/WT*^; *p53*^fl/fl^ mice and *LSL-Kras*^*G12D/WT*^; *p53*^fl/fl^; *LDHB*^*fl/fl*^ mice at the indicated time points. H&E staining of lung tissue sections after 9-week treatment. Scale bar, 20 μm. **d–h** Lung volume **d**, tumor burden **e**, tumor number **f**, average tumor size **g**, and survival fraction **h** after SSZ treatment. Data are shown as the mean ± s.d. *p < 0.05; **p < 0.01; ***p < 0.001 by one-way ANOVA. NS not significant.

We validated our in vitro results in *KRAS*-mutant NSCLC xenografts and in a genetically engineered mouse (GEM) model of *Kras*^*G12D*^-induced NSCLC model, which closely resembles the human disease [10]. In A549 and H460 xenografts, erastin (30 mg/kg) and SSZ (150 mg/kg) significantly and consistently suppressed the growth of LDHB KD tumors but not control tumors, despite no apparent toxicities (Fig. 6a, b; Fig. S6a). Notably, erastin upregulated the lipid peroxidation marker 4-HNE, but not the apoptotic marker caspase-3, in residual LDHB KD tumors, supporting that the in vivo effect of LDHB/SLC7A11 inhibition is driven by ferroptosis (Fig. S6b–d).

Similar results were observed in the GEM model, where SSZ had only mild effects on KP (*Kras*^*G12D/WT*^*; p53*^*fl/fl*^) tumors, but significantly suppressed KPL (*Kras*^*G12D/WT*^*; p53*^*fl/fl*^*; Ldhb*^*−/−*^) tumor growth (Fig. 6c). This was demonstrated by a substantial reduction in tumor burden, as evidenced by preserved lung volume and reduced tumor size and numbers (Fig. 6d–g). Additionally, SSZ-treated KPL mice exhibited a significantly improved survival rate compared to SSZ-treated KP mice (Fig. 6h). These in vivo results corroborate our in vitro data and suggest that LDHB/SLC7A11 inhibition is a viable strategy for targeting *KRAS*-driven NSCLC.

**Fig. 7:**
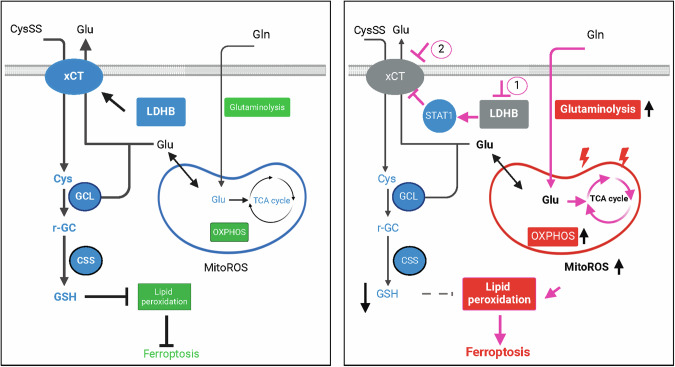
Working model for the function of LDHB in ferroptosis surveillance. LDHB noncanonically protects *KRAS*-driven lung cancer from ferroptosis by promoting the SLC7A11/GSH axis. LDHB blockade is synthetic lethal with SLC7A11 inhibitors due to hyperactivation of glutamine metabolism and mitoROS-dependent ferroptosis.

In conclusion, our in vitro and in vivo results support a model that LDHB noncanonically promotes GSH-dependent ferroptosis defense through SLC7A11. Targeting LDHB and the GSH axis induces synthetic lethality in *KRAS*-driven lung cancer by activating glutaminolysis, elevating mitoROS, and ultimately triggering ferroptosis (Fig. 7).

## Discussion

Here, we provide evidence that the glycolytic enzyme LDHB, but not LDHA, non-canonically promotes the GSH-dependent ferroptosis defense in *KRAS*-driven NSCLC and that inhibition of LDHB and the SLC7A11/GPX4 axis confers ferroptosis-mediated metabolic synthetic lethality.

It is widely accepted that oncogenic KRAS dysregulates metabolism to promote tumorigenesis [7, 47]. A major metabolic manifestation of *KRAS*-driven cancer is the abnormal ROS production [5], although the ROS surveillance mechanisms specifically co-opted by KRAS remain incompletely understood. Here, we report for the first time that LDHB is part of the antioxidant program utilized by *KRAS*-mutant NSCLC to protect against ferroptosis, a ROS-dependent mode of cell death driven by uncontrolled lipid peroxidation [25]. We show that LDHB silencing impairs GSH biosynthesis, which is mediated by the upregulation of STAT1, the transcription factor previously shown to negatively regulate SLC7A11 expression [40], shedding mechanistic light on the critical role of LDHB in this disease [20, 22]. Our findings are consistent with the increasingly appreciated consensus that bypass or silencing of ferroptosis is a hallmark of cancer and that the SLC7A11/GSH axis is a key anti-ferroptosis program co-opted by mutant *KRAS* to overcome the oxidative barrier during tumor development and progression [33, 34, 48–51].

Our finding that STAT1 plays an important role in LDHB-mediated regulation of SLC7A11 is reminiscent of the well-established mechanisms by which immune cell-derived IFNs activate the IFNα/γ-STAT pathway, leading to the suppression of SLC7A11 and promoting ferroptosis in cancer cells [40]. Notably, our previous research has shown that LDHB silencing activates INF response pathways in *KRAS*-mutant NSCLC cells [20], aligning with the protumor activities of IFN within cancer cells [52]. In this study, we show that STAT1 directly regulates *SLC7A11* expression by binding to the GAS2 domain of its promoter, a binding event significantly enhanced by LDHB KD. These findings suggest that LDHB KD may activate IFN signaling, resulting in increased STAT1 expression or enhanced protein stability. Given LDHB’s metabolic role, particularly in lactate metabolism, another plausible mechanism involves changes in specific metabolites following LDHB KD that could modulate STAT1 transcription, translation, or post-translational modifications (PTMs). For instance, PTMs such as lactylation, known to impact gene expression, might contribute to this regulatory network [53, 54]. The non-canonical functions of metabolic enzymes, as described for LDHA, underscores the broader regulatory capacities of these enzymes beyond their conventional metabolic functions [17, 23, 24, 39]. Although further experimental validation is needed, our findings highlight a novel role for LDHB in ferroptosis defense that extends beyond its traditional enzymatic activities.

Given that *KRAS* mutations reprogram cancer metabolism [3, 6], contextually co-opted metabolic dependencies in *KRAS*-mutant cancers have been widely pursued for their therapeutic potential [10, 33, 34, 55–57]. Here, we identify and validate metabolic synthetic lethality through inhibition of the LDHB and GSH-dependent antioxidant program. Mechanistically, inhibition of LDHB and SLC7A11 converges on increased glutamine metabolism, glutaminolysis and OXPHOS, which abnormally upregulates mitochondrial ROS and in turn triggers ferroptosis. Our results are consistent with previous findings that mitochondria play a critical role in cysteine-deprivation-induced ferroptosis [45, 46], that mitochondrial metabolism regulates ferroptosis [58], and that LDHB regulates mitochondrial activity [15, 20], and extend the prior work by demonstrating that the SLC7A11/GSH antioxidant axis is an effector of LDHB and that LDHB/SLC7A11 inhibition induces metabolic synthetic lethality, providing a novel, ferroptosis-based strategy for the treatment of *KRAS*-driven NSCLC.

LDHA/B are subunits of the active LDH enzyme, traditionally recognized for their roles in ATP production and energy homeostasis in both anaerobic and aerobic glycolysis [13]. LDH is crucial for cancer progression, with both LDHA and LDHB being essential for the development of *KRAS*-mutant lung cancer by regulating various aspects of tumor biology [20, 21]. Surprisingly, our findings show that only LDHB, and not the closely related LDHA, promotes ferroptosis defense in *KRAS*-driven NSCLC, highlighting distinct roles for these isoenzymes in the disease. The functional differences between LDHA and LDHB are influenced by several biological factors, including sequence and structural variations, substrate preferences, and distinct metabolic roles. While LDHA and LDHB share sequence and structural similarities, they differ in specific amino acid residues that impact their catalytic properties. LDHA has a higher affinity for pyruvate and is optimized for reducing pyruvate to lactate, supporting anaerobic glycolysis. In contrast, LDHB preferentially catalyzes the conversion of lactate back to pyruvate, feeding into mitochondrial OXPHOS [13]. The role of LDHB in mitochondrial metabolism is particularly significant in *KRAS*-driven cancers, which rely heavily on oxidative metabolism and robust antioxidant systems to maintain redox balance [5–9]. This reliance may explain LDHB’s unique contribution to ferroptosis defense in these cancer cells [20, 45]. In addition, LDHA and LDHB differ in their subcellular localization, which could impact their non-canonical functions [13, 15, 20]. In our study, we demonstrate that LDHB supports redox homeostasis by regulating GSH metabolism, offering new mechanistic insights into its essential role in *KRAS*-driven NSCLC [22]. Future research should explore whether the distinct roles of LDHA and LDHB in redox regulation are conserved across other cancer types. Such investigations may reveal deeper insights into the unique functions of LDH isoenzymes beyond their traditional metabolic activities.

In conclusion, we uncover a *KRAS*-specific mechanism of resistance to ferroptosis. In addition, our results provide strong mechanistic support for combining inhibition of LDHB and the SLC7A11/GSH/GPX4 nexus for the treatment of *KRAS*-driven lung cancer.

## Materials and methods

### Cell culture and reagents

Human NSCLC cell lines (Table S1) were obtained from American Type Culture Collection (Manassas, VA, USA). BEAS-2B and murine KP cells (*Kras*^*G12D*^;*Trp53*^−/−^) were described previously [59]. Cells were cultured in RPMI-1640 medium or Medium 199 (#8758; #4540; Sigma-Aldrich, St. Louis, MO, USA) supplemented with 10% FBS (#10270-106; Life Technologies, Grand Island, NY, USA) and 1% penicillin/streptomycin solution (#P0781, Sigma-Aldrich). All cells were authenticated by DNA fingerprinting and confirmed free from mycoplasma (Microsynth, Bern, Switzerland). Inhibitors were listed in Table S2.

### Cell viability, cell death and clonogenic survival assay

Cells seeded in 96-well plates (2500 cells/well) were dosed 24 h later and cell viability were determined by APH assay as previously described [10, 55]. The efficacy of drugs on cell growth was normalized to untreated control. Each data point was generated in triplicate and each experiment was done three times (*n* = 3). Best-fit curve was generated in GraphPad Prism [(log (inhibitor)) vs. response (-variable slope four parameters)]. Error bars are mean ± SD. Cell death was determined using SYTOX dead cell stain sampler Kit (ThermoFisher Scientific, S34862) according to the manufacturer’s instructions. Clonogenic assay was performed as we described previously [20, 59].

### Gene silencing by small interfering RNA (siRNA), short hairpin RNAs (shRNA) and single-guide RNAs (sgRNA)

Cells at 50-70% confluency were transfected with control or specific pooled siRNA Oligo Duplex (Origene Technologies, Rockville, MD, USA) using Lipofectamine 2000 transfection reagent (Invitrogen, Eugene, OR, USA). Stable knockdown was achieved by lentiviral shRNAs, with lentiviruses produced in HEK293T cells co-transfected with pCMV-VSV-G, pCMV-dR8.2 and shRNA constructs. All shRNAs and siRNAs used in this study are listed in Tables S3 and S4.

### Drug screening

Cells transfected with siLDHB or siNT were re-seeded 24 h later into 96-well plates, treated with the indicated drugs (Table S2) for 72 h before viability assay to determine IC_50_ of each drug in siLDHB and siNT cells. To identify the drugs that differentially affect LDHB KD and siNT cell viability, the IC_10_–IC_20_ dose of each drug in siNT groups (80–90% viability) were used to treat LDHB KD cells. Data analysis, including the calculation of the area under the curve (AUC) was performed using GraphPad Prism 9.1.

### Quantitive PCR, RNA sequencing and analysis

Total RNA was isolated and purified using RNeasy Mini Kit (Qiagen, Hilden, Germany). Complementary DNA was synthesized by the High capacity cDNA reverse transcription kit (Applied Biosystems, Foster City, CA, USA) per manufacturer’s instructions. Real-time PCR (RT-PCR) was performed on a 7500 Fast RealTime PCR System (Applied Biosystems) using TaqMan primer/probes (Table S5**)**. Normalization was based on the ΔΔCT method.

RNA sequencing was performed as previously described [20]. Briefly, sequencing libraries were made using an Illumina TruSeq Stranded mRNA Library Prep kit (#20020595; Illumina) combined with TruSeq RNA UD Indexes (#20022371; Illumina). Pooled cDNA libraries were sequenced paired-end using an Illumina NovaSeq 6000 SP Reagent Kit (#20028401, 100 cycles; Illumina) on an Illumina NovaSeq 6000 instrument. The quality of the sequencing run was assessed using Illumina Sequencing Analysis Viewer (Illumina version 2.4.7) and all base call files were demultiplexed and converted into FASTQ files using Illumina bcl2fastq conversion software v2.20. Pathway enrichment analysis was performed using Metascape (RRID: SCR_016620), and the Gene Set Enrichment Analysis (GSEA) using GSEA software (SeqGSEA, RRID: SCR_005724).

### Immunoblotting, immunohistochemistry and immunofluorescence

Western blot analysis were performed as described [10, 55]. In brief, proteins resolved by SDS-PAGE (#4561033; Bio-Rad Laboratories, Hercules, CA, USA) were transferred onto nitrocellulose membranes (#170-4158; Bio-Rad), which was blocked by blocking buffer (#927-4000; Li-COR Biosciences, Bad Homburg, Germany), incubated with primary antibodies (Table S6) and IRDye 680LT-conjugated goat anti-mouse IgG (#926-68020) and IRDye 800CW-conjugated goat anti-rabbit IgG (#926-32211). Membrane-bound secondary antibodies were imaged using the Odyssey infrared Imaging System (Li-COR Biosciences).

For immunofluorescence, cells grown on poly-lysine-treated coverslides were fixed with 4% paraformaldehyde and permeabilized with 0.1% Triton X-100/PBS before incubated with primary antibodies and appropriate secondary antibodies: Alexa Fluor 647 goat anti-mouse IgG (#A21236) or Alexa Fluor 488 goat anti-Rabbit IgG (#A11034) from Invitrogen. Nuclei were counterstained with DAPI. Images were acquired using a ZEISS Axioplan 2 imaging microscope (Carl Zeiss MicroImaging, Göttingen, Germany) and processed by Adobe illustrator CC 2017 (Adobe Systems, San Jose, CA, USA).

Immunohistochemistry (IHC) was performed as described [10, 55]. In brief, formalin-fixed and paraffin-embedded (FFPE) tumors were sectioned, deparaffinized, rehydrated and stained with hematoxylin and eosin (H&E) and appropriate antibodies (Table S6) using the automated system BOND RX (Leica Biosystems, Newcastle, UK) and visualized by the Bond Polymer Refine Detection kit (Leica Biosystems) as per the manufacturer. Images were acquired by PANNORAMIC® whole slide scanners, processed by Case Viewer (3DHISTECH Ltd.) and quantified by QuPath software.

### Chromatin immunoprecipitation (ChIP) Assay

Chromatin immunoprecipitation was performed using a ChIP assay kit (EMD Millipore 17-295) according to the manufacturer’s protocol. Briefly, one million cells were harvested, and histone DNA was cross-linked with 1% formaldehyde in cell culture medium at 37 °C for 8 min. The formaldehyde was quenched with 125 mM glycine. The DNA of the cells was then sheared to lengths of 200–1000 base pairs using the Bioruptor® Plus sonication device. The sonicated cell supernatants were immunoprecipitated overnight with the STAT1 antibody (Cell Signaling Technology 14994S) or the IgG control antibody after being cleared with protein A agarose/salmon sperm DNA. After three washes of the protein A-agarose/antibody/histone complex, the histone-DNA crosslinks were eluted and recovered by heating at 65 °C for 4 h. DNA was then recovered by phenol/chloroform extraction and ethanol precipitation. Finally, the DNA was analyzed by RT-PCR using specific primers (Table S5).

### Liquid chromatography–mass spectrometry (LC-MS) and metabolomics data analysis

Cells seeded in six-well plates were treated with indicated drugs. Cells were then washed twice with PBS and a solvent (75 mM ammonium carbonate, pH 7.4) and pre-cooled (−20 °C) extraction solvent (40% acetonitrile, 40% methanol, 20% nanopure water) was immediately added to the plates. Cells were then scraped from the dish on the ice, vortexed for 30 s and immediately stocked at –20 °C for 1 h and then at –80 °C. LC–MS measurement and analysis were described previously [60], with metabolomics data analysis with MetaboAnalyst 5.0 software.

### OCR measurements

Cells seeded in Seahorse XF96 V3PS cell culture microplates (Agilent Technologies, 101085-004) were treated, washed twice with PBS and resuspended in Seahorse XF DMEM or RPMI medium (Agilent Technologies, 103680-100, 103681-100), containing 10 mM glucose, 0.5 mM pyruvate, and 2 mM glutamine, with the pH adjusted to 7.4, incubated in a CO_2_-free incubator for 1 h before successively adding 1 μM oligomycin, 1.0 μM and 1.5 μM FCCP, a mixture of 1 μM rotenone and 1 μM antimycin. The data were analyzed using Seahorse Wave (Agilent Technologies). Cell numbers quantification were normalized to 50 ng DNA, which is quantified by CyQUANT™ Cell Proliferation Assay kit (Thermo Fisher Scientific, C7026) according to the manufacturer’s protocol.

### Lipid peroxidation and MitoSOX measurements

Cells in 6-well plates (0.1–0.3 ×10^6^) or 12-well plates (0.05–0.2 ×10^6^) were mixed with 2.5 μM C11_BODIPY (#D3861, Invitrogen) or 5 μM MitoSOX™ Red Mitochondrial Superoxide Indicator (#M36008, ThermoFisher Scientific) followed by flow cytometry (FC) analysis using FACS LSRII instrument (BD Biosciences) to determine lipid peroxidation and mitoROS, respectively. FlowJo V10 was used for data analysis.

### In vivo mouse study

Mouse studies were conducted in accordance with Institutional Animal Care and Ethical Committee-approved animal guidelines and protocols. Xenograft tumors of A549, H838 and H460 cells expressing shLDHB or shNT in NSG (NOD-*scid IL2Rγ*^*null*^) mice were generated and treated as described (31,32), with tumor size calculated as follows: (length × width^2^)/2. *Kras*^*LSL-G12D/WT*^*;p53*^*flox/flox*^*; Ldhb*^*+/+*^ (KP) *and Kras*^*LSL-G12D/WT*^*;p53*^*flox/flox*^; *Ldhb*^*−/−*^ (KPL) mice were described previously [10, 20]. MicroCT images were processed and analyzed using Fiji and 3D Slicer (version 4.13) as we previously described [20]. The sample size was chosen based on standard practice in similar studies to ensure sufficient animals or samples per group for reliable results. Animals were randomly assigned to experimental groups to minimize bias. Tumor induction, drug administration, and outcome assessments (mouse scores, survival monitoring, CT imaging, and tumor measurements) were performed by different investigators to ensure blinding.

### Statistical analysis

Statistical analyses were performed using GraphPad Prism 7.01 (GraphPad Software Inc., San Diego, CA, USA) unless otherwise indicated. In all studies, data represent biological replicates (*n*) and are depicted as mean values ± SD or mean values ± SEM as indicated. In all analyses, *P* values less than 0.05 were considered statistically significant.

## Supplementary information

Supplementary Figures

Supplementary Tables

Original Data File 1

Original Data File 2

Original Data File 3

Original Data File 4

Uncropped WB data

## Data Availability

The RNA sequencing data have been deposited in GEO under the accession number GSE224098. The metabolomics data are available within the Source Data File. All other data are available from the corresponding authors upon request.
