# Supplementary material for: Lactate dehydrogenase B noncanonically promotes ferroptosis defense in KRAS-driven lung cancer
Source: Cell Death Differ. 2024 Dec 7;32(4):632–45. doi: 10.1038/s41418-024-01427-x (PMC11982314; doi:10.1038/s41418-024-01427-x)
Supplement: Supplementary file 1 — Supplementary Figures [file 41418_2024_1427_MOESM1_ESM.docx]

**Supplementary Information**

**Lactate dehydrogenase B noncanonically promotes ferroptosis defense in *KRAS*-driven lung cancer**

Liang Zhao^1,2,11§^, Haibin Deng^1,2,4§^, Jingyi Zhang^1,2^, Nicola Zamboni^5,6^, Haitang Yang^1,2,3^, Yanyun Gao^1,2,7^, Zhang Yang^1,2,8^, Duo Xu^1,2,9^, Haiqing Zhong^1,2^, Gerrit Adriaan Geest^10^, Remy Bruggmann^10^, Qinghua Zhou^7^, Ralph A. Schmid^1*^, Thomas M. Marti^1,2*^, Patrick Dorn^1,2*^, Ren-Wang Peng^1,2*^

^1^Department of General Thoracic Surgery, Inselspital, Bern University Hospital, Bern, Switzerland

^2^Department for BioMedical Research (DBMR), University of Bern, Bern, Switzerland

^3^Department of Thoracic Surgery, Shanghai Chest Hospital, Shanghai Jiao Tong University, Shanghai, China

^4^Second Department of Thoracic Surgery, Hunan Cancer Hospital and The Affiliated Cancer Hospital of Xiangya School of Medicine, Central South University, Changsha, Hunan 410013, China

^5^Department of Biology, Institute of Molecular Systems Biology, Swiss Federal Institute of Technology/ETH Zürich, Zurich, Switzerland

^6^PHRT Swiss Multi-Omics Center, smoc.ethz.ch, Zurich, Switzerland

^7^Lung Cancer Center, West China Hospital, Sichuan University, Chengdu, China

^8^Department of Thoracic surgery, Fujian Medical University Union Hospital, Fuzhou City, Fujian, China

^9^Department of Oncology, The First Affiliated Hospital of Nanjing Medical University, Nanjing, China

^10^Interfaculty Bioinformatics Unit and Swiss Institute of Bioinformatics, University of Bern, Bern, Switzerland

^11^ Present address: Department of Molecular and Integrative Physiology, University of Michigan, Ann Arbor, MI 48109, USA.

^§^Contributing equally to this work

**Running title**: Lactate dehydrogenase B in ferroptosis surveillance

**Keywords:** Lactate dehydrogenase B (LDHB); ferroptosis; SLC7A11; glutathione; glutaminolysis; *KRAS*-dependent cancer

**Conflict of interest:** The authors declare no potential conflicts of interest.

***Corresponding authors:** Ren-Wang Peng ([Renwang.Peng@insel.ch](mailto:Renwang.Peng@insel.ch)); Patrick Dorn ([Patrick.Dorn@insel.ch](mailto:Patrick.Dorn@insel.ch)); Thomas M. Marti ([Thomas.Marti@insel.ch](mailto:Thomas.Marti@insel.ch)); Ralph A. Schmid ([ralph_a_schmid@hotmail.com](mailto:ralph_a_schmid@hotmail.com)).

**
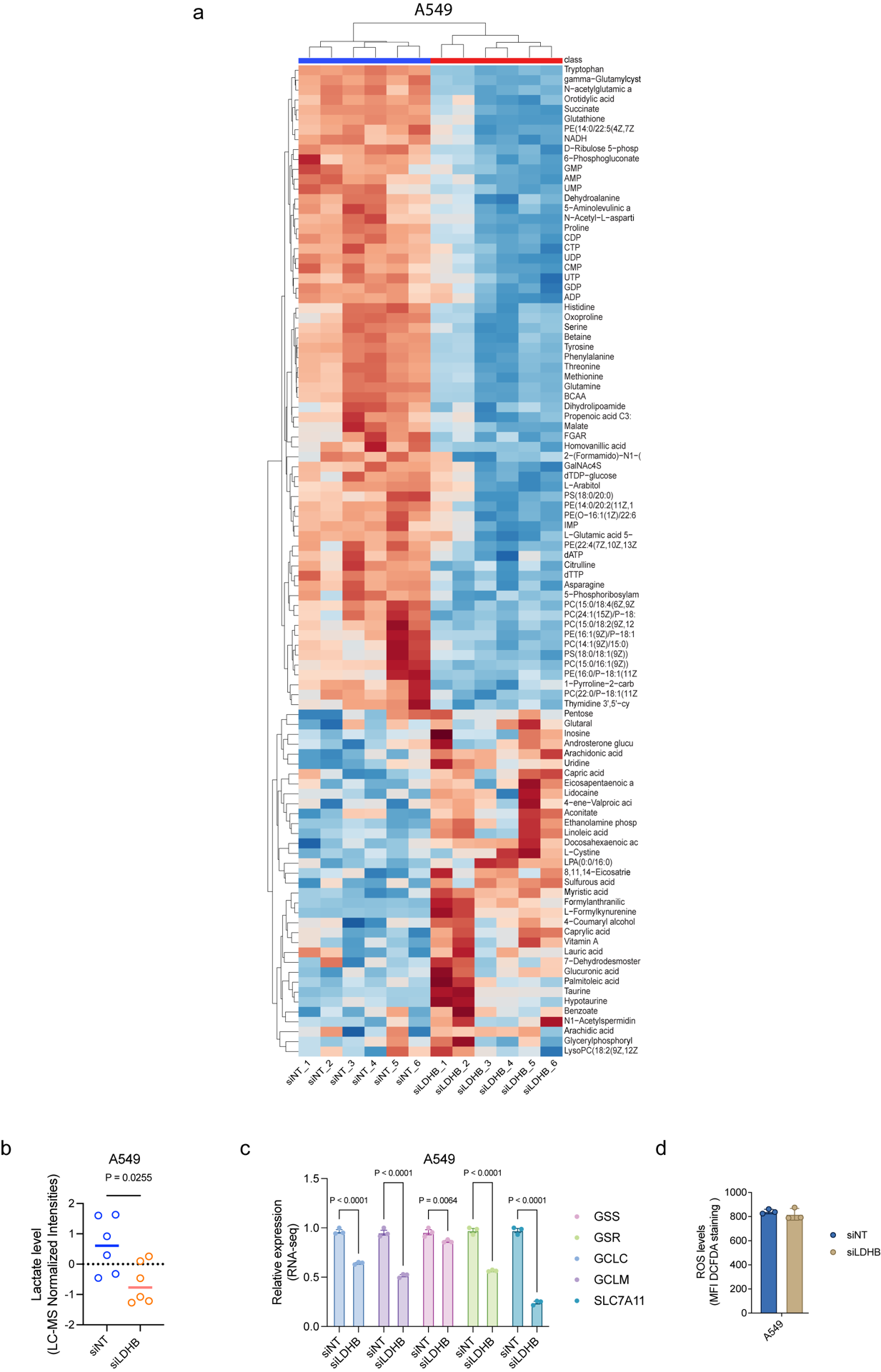
**

**Figure S1,** related to Fig. 1.

**LDHB regulates GSH biosynthesis.**

**a,** Heat maps showing the top 100 significantly different metabolites in siLDHB A549 cells compared to siNT cells. Metabolomics profiling was analyzed 48 h post siRNA transfection.

**b,** The abundance of lactate level in LDHB KD and control A549 cells. Data are analyzed based on the LC-MS analysis and shown as mean ± s.d. (n=3), with *p* values by students’ t test.

**c,** The mRNA levels of key genes involved in GSH synthesis in LDHB KD (48 h post transfection) and control A549 cells. Data are shown as mean ± s.d. (n=3), with p values by students’ t test. GSS: Glutathione synthetase; GSR: Glutathione Disulfide Reductase; GCLC: Glutamate Cysteine Ligase Catalytic Subunit; GCLM: Glutamate Cysteine Ligase Modifier Subunit; SLC7A11: Solute Carrier Family 7 Member 11.

**d,** ROS levels in LDHB KD and control A549 cells. ROS was profiled 48 h post siRNA transfection. Data are shown as as mean ± s.d. (n=3), with p values by students’ t test.


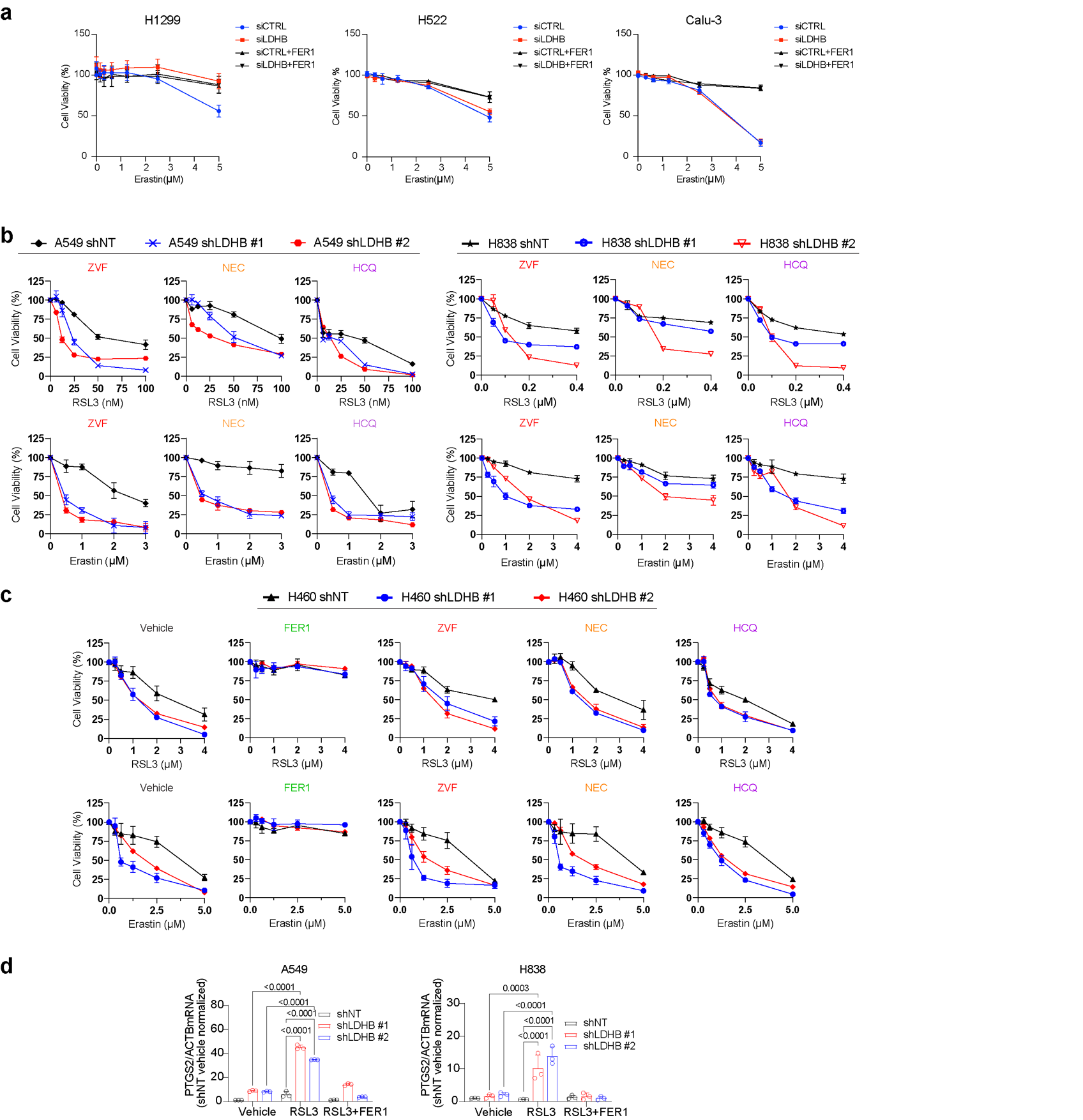


**Fgure S2**, related to Fig. 3.

**LDHB inhibition sensitizes *KRAS*-dependent lung cancer cells to ferroptosis inducers.**

**a,** Viability assay of KRAS wild-type lung cancer cells (H1299, H522, Calu-3) transfected with siNT- or siLDHB-for 24 h and subsequently treated for 48 h with Erastin, alone or in combination with 2 μM Ferrostatin-1(FER1). Data are shown as mean ± s.d. (n=3).

**b-c,** Viability assay of shNT- or shLDHB-transduced cells after treated for 72 h with RSL3 or Erastin, alone or in combination with 2 μM Ferrostatin-1(FER1), 40 μM Z-VAD-FMK(ZVF), 20 μM Necrostatin-1 (NEC1), or 10 μM Hydroxychloroquine (HCQ).

**d,** PTGS2 mRNA levels in A549 and H838 cells after treated with vehicle (DMSO) and RSL3 (0.5 μM), in the presnce or absence of Fer1 (2 μM). Data are shown as mean ± s.d. (n=3), with the statistical analyses by one-way ANOVA. ns, no significant difference.


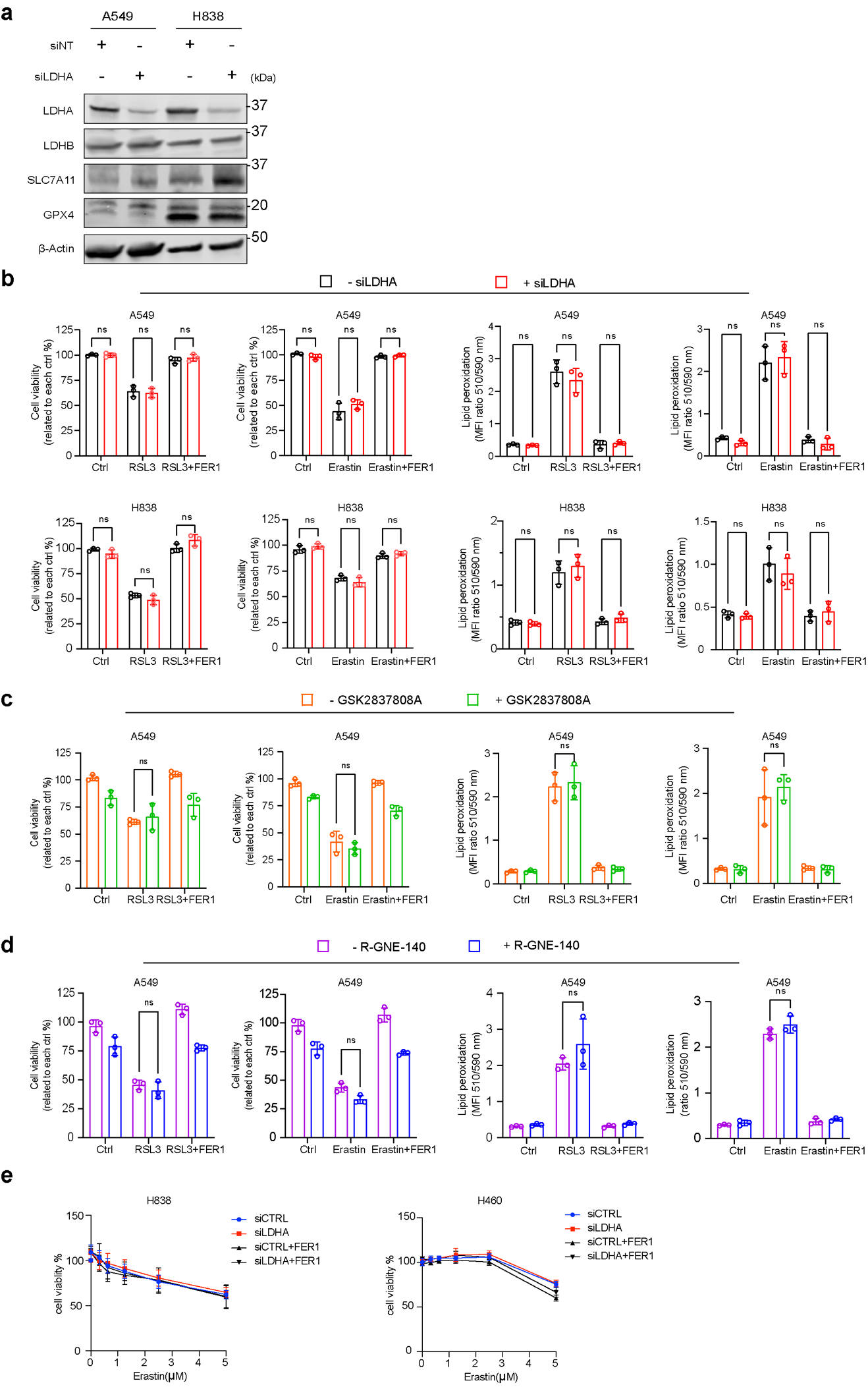


**Figure S3**,

**LDHA is not involved in ferroptosis surveillance in *KRAS*-dependent lung cancer cells.**

**a,** Immunoblot analysis of A549 and H358 cells transfected with siNT or siLDHA for 48 h.

**b,** Viability assay of A549 and H838 cells transfected with siNT or siLDHA and subsequently treated with RSL3 (10 nM), Erastin (1.25 μM) and FER1 (3 μM) for 48 hours, alone and in combination. Lipid peroxidation assay of A549 and H838 cells transfected with siNT or siLDHA and subsequently treated with RSL3 (0.5 μM) for 7 hours and FER1 (3 μM) or Erastin (5 μM) and FER1 (3 μM) for 14 hours, alone and in combination. Lipid peroxidation levels are shown as the mean fluorescence intensity ratio of the oxidative form versus non-oxidative form.

**c,** Viability assay of A549 cells pre-treated with GSK2837808A ( 70 μM) or vehicle, followed by treatment with RSL3 (10 nM) or Erastin (1.25 μM), alone or in combination with FER1 (3 μM) for 48 hours. Lipid peroxidation assay of A549 and H838 cells pre-treated with GSK2837808A ( 70 μM) or vehicle and subsequently treated with RSL3 (0.5 μM) for 7 hours and FER1 (3 μM) or Erastin (5 μM) and FER1 (3 μM) for 14 hours, alone and in combination.

**d,** Viability assay of A549 cells pre-treated with R-GNE-140 ( 20 μM) or vehicle, followed by treatment with RSL3 (10 nM) or Erastin (1.25 μM), alone or in combination with FER1 (3 μM) for 48 hours. Lipid peroxidation assay of A549 and H838 cells pre-treated with R-GNE-140 ( 20 μM) or vehicle and subsequently treated with RSL3 (0.5 μM) for 7 hours and FER1 (3 μM) or Erastin (5 μM) and FER1 (3 μM) for 14 hours, alone and in combination.

**e,** Viability assay of H838 and H460 cells transfected for 24 h with siNT or siLDHA and subsequently treated for 48 h with Erastin (1.25 μM) and FER1 (3 μM) for 48 hours, alone and in combination. Data are shown as mean ± s.d. (n=3), with statistical analyses by two- way ANOVA. ns, no significant difference.

**
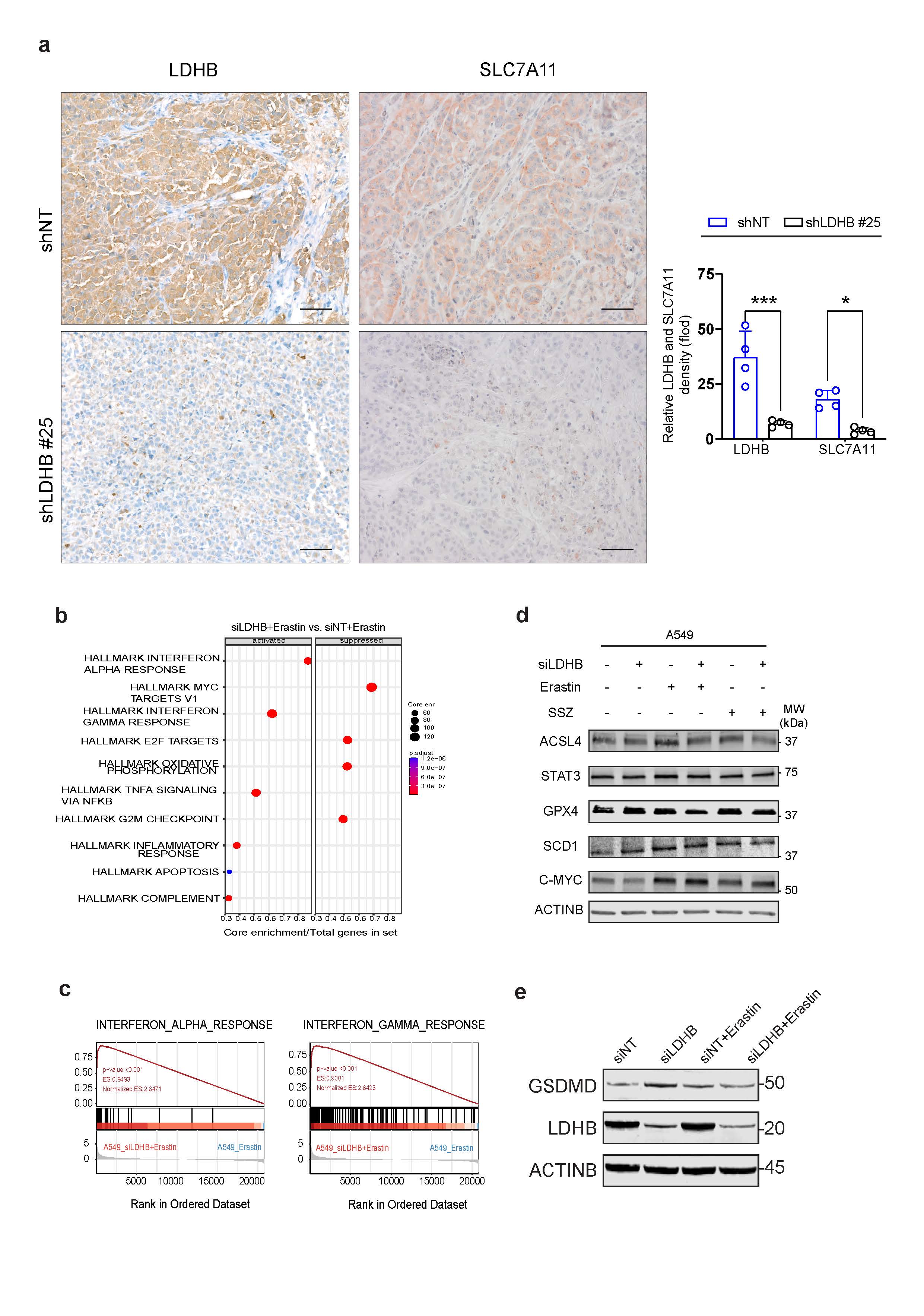
**

**Figure S4**, related to Fig. 4

**LDHB modulates SLC7A11 expression via STAT1.**

**a,** IHC of LDHB and SLC7A11 in LDHD KD and control A549 xenograft tumors, with quantification shown to the right, with **p*<0.01, ****p*<0.001 by students’ t test.

**b,c,** Pathway enrichment and GSEA based on the transcriptome of LDHB KD cells treated with Erastin (siLDHB+Erastin) and of control A549 cells treated with Erastin (siNT+Erastin).

**d,** Immunoblots of ACSL4, STAT3, GPX4, SCD1, and C-MYC in A549 cells after the indicated treatment.

e, Immunoblots of GSDMD and LDHB in A549 cells transfected for 48 h with siLDHB and siNT and subsequently treated for 20 h with or without Erastin.

**
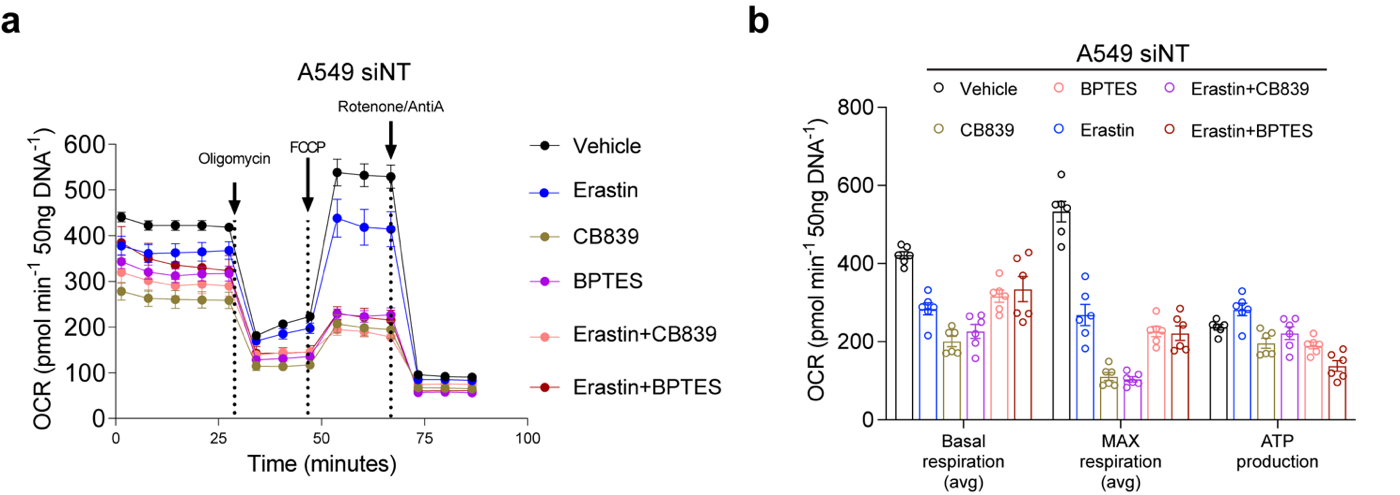
**

**Figure S5, related to Fig. 5**

**a, b**, OCR measure (a) and quantification (b) of A549 KD cells transfected with siNT for 48 h and further treated for 20 h with DMSO, Erastin (5 μM), CB839 (0.5 μM), BPTES (2 μM), and GPNA (50 μM), alone or in combination. Normalization was based on vehicle-treated siNT groups (set as 100%). Avg represents the average of basal respiration, maximal respiration, and ATP production taken at multiple time points during the respective phases of the Seahorse assay. Data are shown as mean ± s.e.m (n=3), with the statistical analyses by one-way ANOVA. ns, no significant difference.


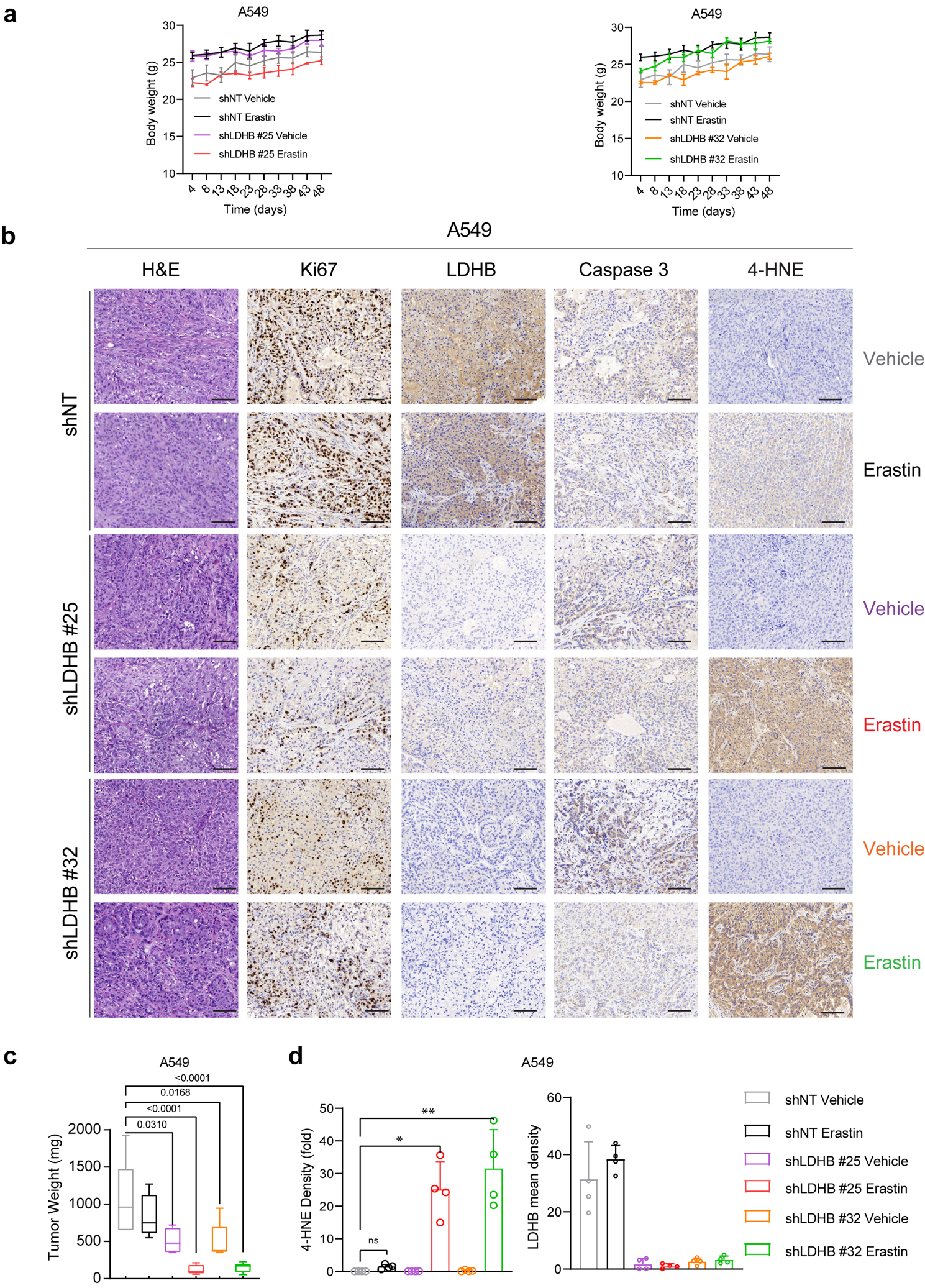


**Figure S6**, related to Fig 6,

**In vivo efficacy of Erastin in LDHB-deficent *KRAS*-driven lung cancer models.**

**a-d,** body weight (a), IHC analysis (b) and tumor weight (c) of xenograft tumors (shNT A549 and shLDHB A549) treated with vehicle or Erastin (30 mg/kg/day). Quantification of IHC staining (c) are shown in (d). Data are shown as mean ± s.d. of three biological repeats (n=3), with the statistical analysis by two-way ANOVA. **P*<0.05, ***P*<0.01, ns, no significance.
