# Supplementary material for: Lactate dehydrogenase B noncanonically promotes ferroptosis defense in KRAS-driven lung cancer
Source: Cell Death Differ. 2024 Dec 7;32(4):632–45. doi: 10.1038/s41418-024-01427-x (PMC11982314; doi:10.1038/s41418-024-01427-x)
Supplement: Supplementary file 2 — Supplementary Tables [file 41418_2024_1427_MOESM2_ESM.docx]

**Table S1. Cell lines used in this study**

| **Cell**  **lines** | **Tissue** | ***KRAS*** | ***STK11*** | ***KEAP1*** | ***NFE2L2*** | ***TP53*** | ***PIK3CA*** | ***BRAF*** | ***EGFR*** |
| --- | --- | --- | --- | --- | --- | --- | --- | --- | --- |
| A549 | Lung | G12S | Nonsense | Mis | WT | WT | WT | WT | WT |
| H838 | Lung | AMP | WT | Mut | WT | Mis | WT | WT | WT |
| H460 | Lung | Q61H | Nonsense | Mis | WT | WT | Mis | WT | WT |
| H2122 | Lung | G12C | DEL | Mis | WT | Mis | WT | WT | WT |
| PC9 | Lung | WT | WT | WT | WT | Mut | WT | WT | MUT |
| AsPC-1 | Pancreas | G12D | WT | WT | WT | Del | WT | MUT | WT |
| HT1080 | Connective tissue | WT | WT | WT | WT | WT | WT | WT | WT |
| BEAS2B | Normal | WT | WT | WT | WT | WT | WT | WT | WT |
| KP | Mouse Lung | G12D | WT | WT | WT | Mut | WT | WT | MUT |
| H1299 | Lung | WT | WT | WT | WT | WT | WT | WT | WT |
| H522 | Lung | WT | WT | WT | WT | Mut | WT | WT | WT |
| Calu-3 | Lung | WT | WT | WT | WT | Mut | WT | WT | WT |

**Table S2. Compounds used in this study**

| **Pathway** | **Drug** | **Target** | **Company** | **Cat. #** |
| --- | --- | --- | --- | --- |
| RTK | AZD4547 | FGFR | Selleckchem | S2801 |
|  | Afatinib | EGFR | Selleckchem | S1011 |
|  | Neratinib | EGFR | Selleckchem | S2150 |
| PI3K/AKT/mTOR | LY294002 | PI3K | Selleckchem | S1105 |
|  | AZD5363 | AKT | Selleckchem | S8019 |
|  | Rapamycin | MTOR | Selleckchem | S1039 |
| RAF/MEK/ERK | sorafenib | Raf/SLC7A11 | Selleckchem | S1040 |
|  | Trametinib | MEK | Selleckchem | S2673 |
| Synthetic Lethality | Dasatinib | SCR | Selleckchem | S1021 |
|  | Palbociclib | CDK | Selleckchem | S4482 |
|  | QNZ | NFkB | Selleckchem | S4902 |
|  | Onalespib | HSP90 | Selleckchem | S1163 |
|  | Bortezomib | Proteason | Selleckchem | S1013 |
|  | BI 2536 | BET/PLK1 | Selleckchem | S1109 |
| Metabolism related | FK866 | NAMPT | Selleckchem | S2799 |
|  | VLX600 | OXPHOS | Selleckchem | S8943 |
|  | GBOXIN | OXPHOS | Selleckchem | S8828 |
|  | CB839 | GLS | Selleckchem | S7655 |
|  | C75 | FASN | Selleckchem | S9819 |
|  | CBR5884 | ATP | Selleckchem | S9645 |
|  | ACIVICIN | GGT | Chemscene | CS-W017302 |
|  | Erastin | SLC7A11 | Chemscene | CS-1675 |
| DNA damage | KU57788 | DNAPK | Chemscene | CS-0034 |
|  | Olaparib | PARP | Chemscene | CS-0075 |
| Epigenetic related | GSK126 | EZH2 | Chemscene | CS-1401 |
|  | Vorinostat | HDAC | Chemscene | CS-0589 |
| JAK/STAT | Ruxolitinib | JAK1/2 | Selleckchem | S1378 |
| others | GSK2837808A | LDHA | MedChem Express | HY-100681 |
|  | (R)-GNE-140 | LDHA | Selleckchem | S6675 |
|  | Sulfasalazine | SLC7A11 | MedChem Express | HY-14655 |
|  | RSL3 | GPX4 | Selleckchem | S8155 |
|  | ML162 | GPX4 | Selleckchem | S4452 |
|  | Tofacitinib | JAK1/2/3 | Selleckchem | S5001 |
|  | GPNA  (hydrochloride) | GLS | Chemscene | CS-W012107 |
|  | BPTES | GLS | Selleckchem | S7753 |
|  | Ferrostatin-1 |  | Chemscene | CS-0019733 |
|  | Liproxstatin-1 |  | MedChem Express | HY-12726 |
|  | Z-VAD-FMK |  | Selleckchem | S7023 |
|  | Hydroxychloroquine Sulfate |  | Selleckchem | S4430 |
|  | Necrostatin-1 |  | Selleckchem | S8037 |

**Table S3. shRNAs and siRNAs used in this study**

| **Product Description** | **Company** | **Cat. #** |
| --- | --- | --- |
| Lactate Dehydrogenase B (LDHB) Human shRNA Plasmid Kit | Origene | TL311768 |
| Ldhb Mouse shRNA Plasmid | Origene | TR513943 |
| pLenti CMV-SLC7A11-sh926R-FLAG-IRES-Hygro | Addgene | 118702 |
| Trilencer-27 Universal scrambled negative duplex | Origene | SR30004 |
| Lactate Dehydrogenase B (LDHB) Human siRNA Oligo Duplex | Origene | SR320835 |
| xCT (SLC7A11) Human siRNA Oligo Duplex | Origene | SR308432 |
| STAT1 Human siRNA Oligo Duplex | Origene | SR321905 |

**Table S4. Sequences of the shRNA and siRNA used in this study**

| Oligos | Type/Target Gene |
| --- | --- |
| 5’-GCACTACCAGAGCTAACTCAGATAGTACT -3’ | shNT |
| 5’-TGAATGTGGCAGGTGTTTCTCTCCAGGAA -3’ | shLDHB 1 |
| 5’-AGTCTCTGGCTGATGAACTTGCTCTTGTG-3’ | shLDHB 2 |
| 5’-ACCTAAGCACCGTGTGATTGGAAGCGGAT-3’ | shLdhb (mouse) A |
| 5’-CTGGTGGATGTGTTGGAAGACAAGCTCAA-3’ | shLdhb (mouse) B |
| 5’-AAGUACAGUCCUGAUUGCAUCAUAA-3’ | siLDHB_A |
| 5’-UAUGAAGUCAUCAAGCUAAAAGGAT-3’ | siLDHB_B |
| 5’-GAGCCUUUAGUUUUCAUCCAUGUAC-3’ | siLDHB_C |
| 5’-GGAAGACAUCGAUCAGUAACACCAA-3’ | siSLC7A11_A |
| 5’-GCAUGUUAAAGCUGUAUAAUUUGTT-3’ | siSLC7A11_B |
| 5’-GGAACUUACAAAUGAGAGUAAUATA-3’ | siSLC7A11_C |
| 5’-CUUGACAGUAAAGUCAGAAAUGUGA-3’ | siSTAT1_A |
| 5’-ACUCAAGAAGAUGUAUUUAAUGCTT-3’ | siSTAT1_B |
| 5’-ACAGAAAGAGCUUGACAGUAAAGTC-3’ | siSTAT1_C |
| 5’-CGUUAAUCGCGUAUAAUACGCGUAT-3’ | siNT |

**Table S5. Primer sequences for PCR used in this study**

| **Genes** | **Primer sequences** | **Company** |
| --- | --- | --- |
| SLC7A11 (human)-For | 5’ CCTCTATTCGGACCCATTTAGT 3’ | ID Technology |
| SLC7A11 (human)-Rev | 5’ CTGGGTTTCTTGTCCCATATAA 3’ |  |
| PTGS2 (human)-For | 5’ CTGGCGCTCAGCCATACAG 3’ | ID Technology |
| PTGS2 (human)-Rev | 5’ CGCACTTATACTGGTCAAATCCC 3’ |  |
| LDHB (human)-For | 5’ TGGTATGGCGTGTGCTATCAG 3’ | ID Technology |
| LDHB (human)-Rev | 5’ TTGGCGGTCACAGAATAATCTTT 3’ |  |
| B2M (human) -For | 5’ GAGGCTATCCAGCGTACTCCA 3’ | ID Technology |
| B2M (human)- Rev | 5’ CGGCAGGCATACTCATCTTTT 3’ |  |
| ActinB (human) -For | 5’ CATGAAGATCAAGATCATCGCC 3’ | ID Technology |
| ActinB (human)- Rev | 5’ ACATCTGCTGGAAGGTGGACA 3’ |  |
| xCT(GAS2)-For | 5’ TCAGCTTCCTCATGGGCTTG 3’ | Microsynth |
| xCT(GAS2)-Rev | 5’ TGAGCAACAAGCTCCTCCTG 3’ | Microsynth |

**Table S6. Primary antibodies used in this study**

| **Antibody for WB** | **Host** | **Company** | **Cat#** | **Dilution** |
| --- | --- | --- | --- | --- |
| Keap1 (D6b12) | Rabbit | CST | 8047S | 1:1000 |
| γ-GCSc | Mouse | SANTA CRUZ | sc-390811 | 1:500 |
| γ-GCSm | Mouse | SANTA CRUZ | sc-55586 | 1:500 |
| ACSL4 | Mouse | SANTA CRUZ | sc-271800 | 1:500 |
| Xct/slc7a11 (D2M7A) | Rabbit | CST | 12691S | 1:1000 |
| LDHA | Rabbit | CST | 2012 | 1:1000 |
| LDHB | Rabbit | R&D Systems | MAB9205 | 1:20000 |
| LDHB | Rabbit | Proteintech | 14824-1-AP | 1:10000 |
| GPX4 | Rabbit | Abcam | ab125066 | 1:1000 |
| STAT1 | Mouse | CST | 9176S | 1:1000 |
| p-STAT1 (58D6) | Rabbit | CST | 9167S | 1:1000 |
| STAT3 | Mouse | CST | 9139 | 1:1000 |
| p-STAT3 (D3A7) | Rabbit | CST | 52075S | 1:1000 |
| TFRC/CD71 | Mouse | SANTA CRUZ | sc-32272 | 1:400 |
| AKR1C | Mouse | Novus Biologicals | 74042 | 1:500 |
| SCD1 | Rabbit | CST | 2794S | 1:1000 |
| C-MYC | Rabbit | CST | 13987S | 1:1000 |
|  |  |  |  |  |
| beta-Actin (8H10D10) | Mouse | CST | 3700S | 1:5000 |
| beta-Actin (13E5) | Rabbit | CST | 4970S | 1:1000 |
|  |  |  |  |  |
| **Antibody for IHC** |  |  |  |  |
| 4HNE | Rabbit | Abcam | ab46545 | 1:100 |
| Ki-67 | Rabbit | CST | 9027 | 1:400 |
| Caspase 3 | Rabbit | CST | 9664 | 1:2000 |
| LDHB | Rabbit | R&D Systems | MAB9205 | 1:20000 |
| LDHB | Rabbit | Proteintech | 14824-1-AP | 1:10000 |
