# Supplementary figures and images for: Lactate dehydrogenase B noncanonically promotes ferroptosis defense in KRAS-driven lung cancer
Source: Cell Death Differ. 2024 Dec 7;32(4):632–45. doi: 10.1038/s41418-024-01427-x (PMC11982314; doi:10.1038/s41418-024-01427-x)

Fig2a

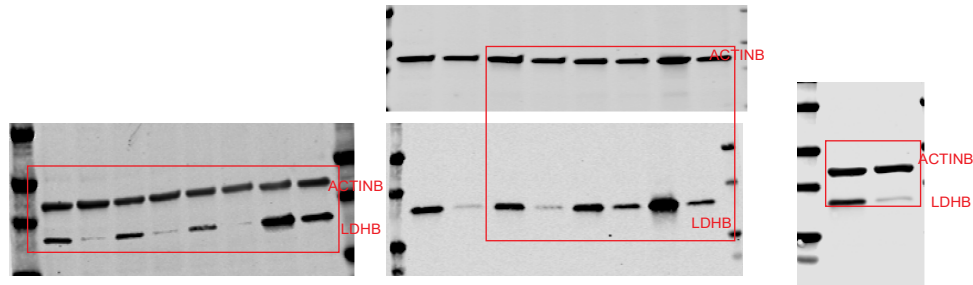

Fig3a

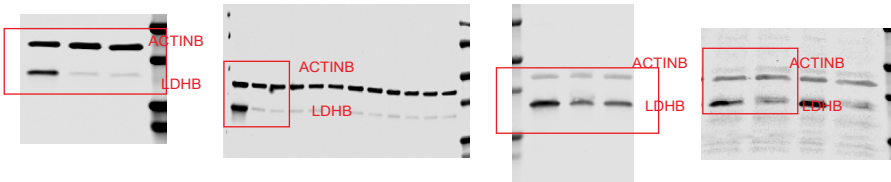

Fig.4b

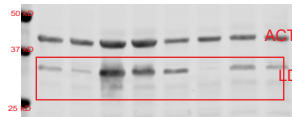

Fig.4c

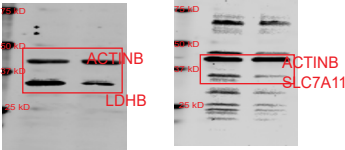

Fig.4d

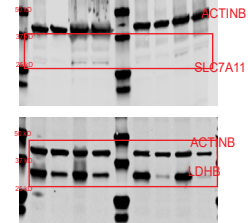

Fig.4g

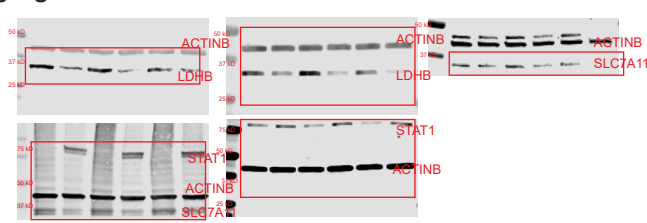

Fig.4h

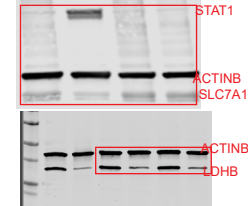

Fig.S3a

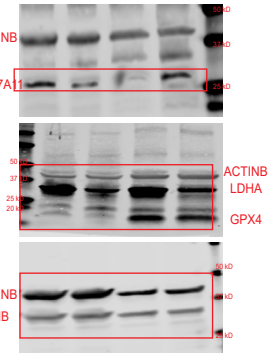

Fig.S4d

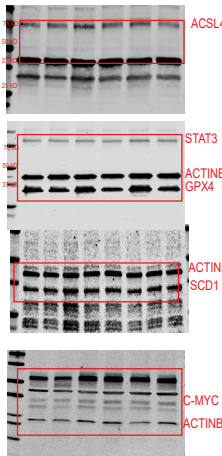

Fig.S4e

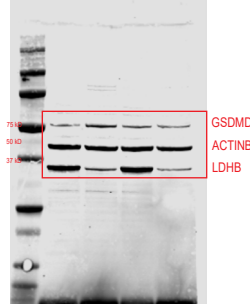

Supplement: Supplementary file 7 — Uncropped WB data [file 41418_2024_1427_MOESM7_ESM.pdf]
